# Effect of Technology and Digital Media Use on Adolescent Health and Development: Protocol for a Multimethod Longitudinal Study

**DOI:** 10.2196/50984

**Published:** 2023-09-13

**Authors:** Christopher N Cascio, Ellen Selkie, Megan A Moreno

**Affiliations:** 1 School of Journalism and Mass Communication University of Wisconsin-Madison Madison, WI United States; 2 Department of Pediatrics School of Medicine and Public Health University of Wisconsin-Madison Madison, WI United States

**Keywords:** social media, adolescents, teenagers, health, well-being, risk behavior, brain, functional magnetic resonance imaging, fMRI, mobile phone

## Abstract

**Background:**

Technology and digital media (TDM) use is integral to modern adolescence; adolescents have been labeled as “digital natives,” since they have had exposure to digital technology for their entire lives. Previous evidence has illustrated TDM’s connections with adolescent risk behaviors such as increased alcohol use and social media exposure, as well as relationships with adolescent well-being such as improved socioemotional health and social media connections with peers. Although several recent review articles have described both the benefits and risks of technology use, most individual studies adopt a singular risk-centered approach. In addition, reviews suggest that little evidence exists on the potential mediating and moderating factors between TDM use and well-being and health outcomes, which limits our understanding of what influences the outcomes of interest. Therefore, there is an urgent need to fill these gaps.

**Objective:**

This protocol addresses the need to understand how TDM exposure and use affect multiple developmental domains and health outcomes. We address the fragmented nature of previous research, the common focus on single behaviors or conditions, and the typical narrow lens on risks. Our approach further aligns with reviews that called for studies identifying and investigating the factors that moderate the relationships between social media and health behaviors and outcomes.

**Methods:**

We will address our objective by longitudinally examining over a 2-year period a common set of adolescent participants (N=400, aged 13-15 years) across 3 studies that adopt a multimethodological approach. Study 1 will use TDM to understand the mechanisms behind adolescent health and risk behaviors. Study 2 will use functional magnetic resonance imaging to understand how positive and negative TDM experiences relate to mental and behavioral health in a subsample of 150 adolescents. Study 3 will use a mixed methods design to evaluate self- and other-generated TDM content as the predictors of socioemotional well-being in sexual and gender minority and non–sexual and gender minority adolescents.

**Results:**

Recruitment is ongoing, and the initial results from the first wave of recruitment are expected in 2024.

**Conclusions:**

This integrated approach to longitudinal data collection from a shared adolescent participant pool will lead to novel analyses and findings, allowing for the examination of the health and well-being risks and benefits associated with TDM use and factors that moderate these relationships. The findings from this study will advance conceptual models and inform new interventions to improve adolescent health.

**International Registered Report Identifier (IRRID):**

DERR1-10.2196/50984

## Introduction

### Background

Technology and digital media (TDM) use is integral to modern adolescence; adolescents have been labeled as “digital natives,” since they have had exposure to digital technology for their entire lives [1-3]. The 2022 Pew Internet and American Life Project [4] estimated that 95% of US adolescents have their own personal smartphone. These rates have increased from 2014 to 2015, when 73% of adolescents reported personal smartphone access [5]. Moreover, teenagers have begun obtaining their first phone at a younger age, with most adolescents owning a phone by the age of 13 years [6,7]. The nearly ubiquitous and thus dominant form of TDM use among adolescents is social media use. Web 2.0 led to what has been called social media, which is also called immersive or interactive media.

Social media represents a set of Web 2.0 tools that are centered on interaction and sharing of content with others, although users may also consume content passively. In the world of social media, adolescents have become both creators of self-generated content and consumers of other-generated content. Messages and content can flow in all directions, from peers to other users, among other users, and across platforms through a seemingly endless array of potential paths. Social media is used for a variety of activities, including sharing information, interacting with peers, and developing an identity [8]. Most adolescents report the ownership of at least 1 social media account or profile, and most maintain them on several platforms [9]. Thus, today’s adolescents have an increased capacity to interact with each other and the larger world using media, enhanced opportunities to explore and experiment via media, and an increased likelihood of being influenced by media experiences. The recent social isolation and disrupted school schedules experienced by adolescents during the COVID-19 pandemic have highlighted the diverse uses and values of TDM for adolescents [10].

Given that adolescents’ development includes identity development, peer relationships, and independence, the tools offered by social media seem almost perfectly aligned with this period [11]. Research supports the idea that youths use TDM to achieve critical developmental tasks [12], such as seeking a coherent sense of self via self-presentation and experimenting with identity. This social media–based identity development may be even more important for adolescents who identify as part of minority groups, such as the lesbian, gay, bisexual, trans, queer, or intersex community [13,14], and for adolescents with chronic illnesses [15]. Further, social media can offer the opportunity to engage in aspirational development, providing a place to reflect on, complement, and reinforce offline relationships [16], practices, and behaviors [17]. Social media combines peer and media effects and thereby represents a powerful motivator of behavior, whether through self-generated or other-generated (ie, consumed) content. Social media brings together the power of interpersonal persuasion with the reach of mass media and led to a new concept of “mass interpersonal persuasion,” which is “the most significant advance in persuasion since radio was invented in the 1890s” [18].

Many studies on adolescent TDM use have focused solely on risks and negative outcomes. Previous studies have illustrated that TDM use is associated with negative outcomes such as impaired sleep [19-21], decreased physical activity [20,22,23], problematic internet use [24-26], and risk for depression [27]. Although several recent review articles have described both the benefits and risks of technology use [8,28], most individual studies adopt a singular risk-centered approach [29,30]. The benefits of social media include increased social capital [31], safe identity exploration [32], social support, and an opportunity for self-disclosure [33]. Specifically, in the area of adolescent health, previous studies have shown that TDM offers opportunities to provide novel interventions for pediatric patients [34], advance reproductive and sexual health [35,36], and provide new approaches to mental health support [37]. Those who experience isolation, stress, and unmet needs in their offline worlds may find corrective experiences or buffering effects by going on the web [38,39]. For example, a qualitative interview study found that young people naturally and organically developed close-knit communities of close friends, often in the form of private Instagram (Meta Platforms, Inc) accounts, in which privacy was a priority and emotional disclosure was safe and commonplace [40].

### This Protocol

#### Overview

In this protocol, we will address the fragmented nature of previous research, the common focus on single behaviors or conditions, and the typical narrow lens on risks. Although scholars have called for comprehensive approaches considering the benefits and risks of technology use [8,28], most existing studies focus on risks [29,30]. Our approach is aligned with a previous review that called for studies identifying and investigating the factors that moderate the relationships between social media and health behaviors and outcomes [8]. This approach is further highlighted in the 2023 US Surgeon General’s Advisory on Social Media and Youth Mental Health, which calls for research examining the benefits and risks associated with specific social media designs, features, and content [41]. Studies in this protocol will incorporate a particular emphasis on 2 key attributes of adolescent development: identity development and social development.

#### Identity Development

Social media offers the opportunity for identity exploration and development via the creation of personal profiles and the sharing of self-generated content. A previous study found that adolescents who communicated more on the web had greater self-concept clarity, the ability to understand oneself clearly and stably [42]. For adolescents using social media to explore their identity, social media may provide a conducive environment for receiving positive or negative social feedback on their identity. Another aspect of web-based identity development is impression management [43]: adolescents use social media for impression management by using their self-representation to control or influence other people’s perceptions of who they are and how they act. In all 3 studies in this protocol, evaluations will incorporate adolescents’ self-generated content on social media.

#### Social Connection

Social connectedness and inclusion are essential for well-being during adolescent development. Social media offers adolescents the capacity to build peer connections by sharing content, endorsing each other’s content, and communicating publicly and privately. By viewing their peers’ content, adolescents may engage in social comparison [44] and potentially emulate behaviors or internalize the ideas seen in social media posts. Other-generated content may thus be persuasive through both peer and media effects. In all 3 studies in this protocol, we will incorporate evaluations of other-generated content consumed by adolescents via TDM.

## Methods

### Project Overview

Our overall objective for the 3 studies in this protocol is to understand the aspects of behavior and socioemotional well-being, alongside neurocognitive correlates, and how self-generated (ie, created) and other-generated (ie, consumed) social media content influence and associate with them. This comprehensive approach to investigation across multiple dimensions of health and technology use focuses on an integrated research strategy and interdisciplinary expertise. This protocol describes a program of research across 3 studies that leverage a shared participant pool and data to examine the health risks and benefits associated with social media use over a 2-year period during adolescence. Our long-term goal is to use the findings from this synergistic protocol to enhance health messaging campaigns, improve big data surveillance approaches, develop innovative interventions, and advance conceptual models. This program protocol includes the following 3 studies.

### Study 1

Study 1 will focus on TDM use to understand the mechanisms behind adolescent health and risk behaviors. Health behaviors will include behaviors that advance adolescent physical and mental health, including physical activity and sleep. Risk behaviors will focus on substance use, including the use of 10 categories of substances most often used by adolescents (eg, alcohol, tobacco, and marijuana). Substance use is connected with the major causes of morbidity and mortality in adolescents: accidents (particularly motor vehicle accidents), homicide, and suicide. Study 1 will include a 2-year data collection process, wherein social media data created by adolescents will be triangulated with self-reported health attitudes, social norms, intentions, and behaviors (aim 1). Study 1 will also include an assessment of other-generated TDM content reported by adolescents via an ecological momentary assessment (EMA) approach to capture real-time exposure to health and risk behavior content on TDM (aim 2). This study will also examine the persuasive elements of self-generated and other-generated content via functional magnetic resonance imaging (fMRI; aim 3). This project will include technology importance and parent involvement as moderators.

### Study 2

Study 2 will use fMRI to understand how neural correlates associated with positive and negative TDM experiences relate to mental and behavioral health. Drawing on social media use data on health and risk behaviors and well-being, from studies 1 and 3, study 2 will assess reactivity (ie, neural activity) in reward-processing regions during social inclusion and reactivity in social pain regions during social exclusion at 2 time points, during middle school (time 1; 13 and 14 years of age) and after the transition to high school (time 2; 15 and 16 years of age). For study 2, aim 1 will include fMRI data collection at time 1 to examine whether the main effects of reactivity to social inclusion and exclusion predict health and risk behaviors and well-being at times 1 and 2. Aim 2 will include fMRI data collection at time 1 to examine whether neural reactivity to social inclusion and exclusion moderates the relationship between social media use (ie, likes, comments, number of friends or followers) and health and risk behaviors and well-being at times 1 and 2. Aim 3 will include fMRI data collected at times 1 and 2 to examine whether changes in reactivity to social inclusion and exclusion are associated with changes in one’s social media environment (ie, positive valence peer interactions vs negative valence peer interactions) during the transition from middle school to high school, health and risk behaviors, and well-being, including technology importance and parent involvement as moderators, at time 2.

### Study 3

Study 3 will use mixed methods to evaluate self- and other-generated TDM content as predictors of socioemotional well-being in sexual and gender minority (SGM) and non-SGM adolescents. Socioemotional well-being will include concepts of self-esteem, loneliness, stress, emotional difficulties, and subjective well-being, all of which are contributors to the overall mental health and subsequent morbidity and mortality in adolescents. Study 3 will involve data collection over 2 years to test a cross-lagged panel model of the relationships among self-generated social media content (aim 1), other-generated social media content (aim 2), and socioemotional well-being. Socioemotional well-being will be assessed through validated self-report instruments at baseline, 1 year, and 2 years. Self-generated social media content will be assessed using ethnographic observation and coding of participants’ social media accounts for common patterns to create latent variables of self-generated content types. Other-generated content will be assessed through the direct observation of social media profiles that are followed by participants, which will be coded for common patterns and triangulated with participant interview data to create latent variables of other-generated content types. Study 3 will also examine self-generated and other-generated social media content of SGM participants, which will be qualitatively compared with the social media content of non-SGM participants (aim 3). SGM adolescents are at an increased risk for threats to socioemotional well-being and are also high-frequency users of TDM, warranting additional exploration of their social media experiences.

### Study Design

**Figure 1:**
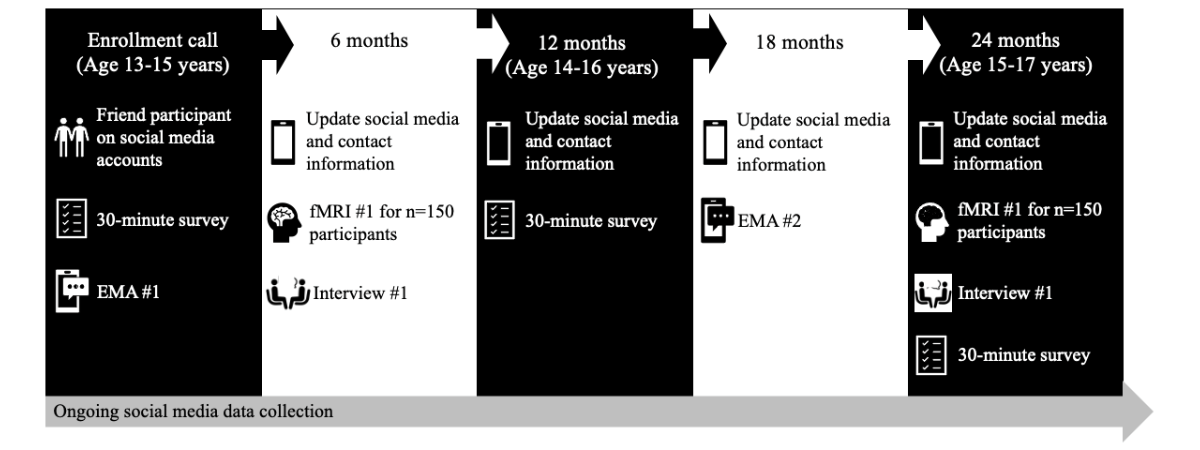
Study activities and participant experience timeline. On the basis of the shared participant pool, synergy in measures, and integration of data collection approaches, participants will have the same study experience even when starting at different time points. EMA: ecological momentary assessment; fMRI: functional magnetic resonance imaging.

This multimethodological protocol will recruit 400 adolescents aged 13 to 15 years to take part in a 2-year study on a rolling basis. Eligible participants will have at least 1 active social media account (ie, Instagram, Facebook [Meta Platforms, Inc], Twitter [Twitter, Inc], or TikTok [ByteDance]). A subsample of 150 participants who meet the fMRI criteria will be recruited to participate in a 2–time point fMRI study in addition to the primary study. After gaining written informed assent from participants and consent from a parent or guardian, researchers will friend the social media accounts of participants to track their social media data over the 2-year enrollment period. Researchers will conduct a check-in at 6, 12, 18, and 24 months to update the research team on the current social media platform use. In addition, participants will complete a series of web-based self-report measures regarding health behaviors, well-being, and demographic information at the beginning of the study (0 mo), 12 months, and 24 months. Participants will also complete an EMA about health behaviors and social media content exposure at 0 and 18 months. Finally, participants will complete interviews at 6 and 24 months about their well-being and social media use. Participants enrolled in the fMRI portion of the study will also be asked to complete neuroimaging appointments at 6 and 24 months to collect neural responses associated with social connection (ie, self- vs other-generated social media posts, peer feedback, social influence, and social inclusion and exclusion) and self-reported individual difference measures and health behaviors. The study activities and participant experience timeline are summarized in Figure 1.

### Participants

#### Inclusion and Exclusion Criteria

Our inclusion criteria will include 400 adolescents living in Wisconsin who are aged 13 to 15 years at enrollment. Adolescents must own and maintain at least 1 social media profile on any of the social media platforms covered in our study (Facebook, Instagram, Twitter, and TikTok) at the time of enrollment. A social media profile will be defined as an individual (not a group or an organization) profile that the adolescent logs into and interacts with at least weekly. Participants must be able to speak, post content, and read in English.

A total of 150 participants (aged 13 and 14 years) from the 400 participants in the shared participant pool will be invited to take part in fMRI data collection. All fMRI participants will be right handed, not have claustrophobia, not currently be taking any psychoactive medications, have no history of psychiatric or neurological disorders, have normal (or corrected to normal) vision, not have metal in their body that is contraindicated for fMRI, and not currently be pregnant. Finally, participants should be in eighth grade at the time of the first scanning appointment to examine whether the social transition into high school alters neural function during development.

#### Recruitment and Enrollment

Participants will be recruited using a variety of methods (eg, on the web, mailers, recruitment ambassadors, speaking events, and pediatric clinics) from the state of Wisconsin, with efforts focused on the regions of and surrounding Madison, Green Bay, Beloit, and Milwaukee. To achieve our recruitment goal of including participants who represent communities of interest, we will use a quota sampling approach [45-47] and prescreen potential participants before enrollment. Screening will involve a brief demographic survey assessing age, race, ethnicity, gender identity, socioeconomic status (SES), and the identified sexuality. After screening, we will assess whether participants meet the criteria for recruitment based on the availability within each study quota. If a participant meets the recruitment criteria, we will proceed with consent and assent and recruitment.

The current protocol aims to recruit a sample reflective of the demographics of the United States in terms of sex, race, ethnicity, and SES. These demographics include, as of 2020, sex (50% female), ethnicity (18.7% Hispanic and Latino), and race (61.6% White, 12.4% Black, 6% Asian, 1.3% American Indian and Native Hawaiian or Pacific Islander, and 10.2% more than 1 race) [48]. Regarding SES, we will ask at screening whether an adolescent has free or reduced-price lunch at school. This proxy measure for SES will be useful for adolescents who are screened in the absence of a parent and may not know their household income. From 2019 to 2020, 52.1% of the students were eligible for free or reduced-price lunch [49]. Regarding SGM status, given study 3’s focus on SGM social media activities (aim 3), we will oversample individuals who identify as SGM. To reach theoretical saturation for qualitative analyses, we aim to recruit a minimum of 30 gender minority youths and 50 sexual minority youths. These estimates are based on our team’s prior experiences with qualitative sampling.

### Sample Size Calculations

Separate sample size calculations were performed for each of the 3 studies in this protocol based on the primary analyses that will be implemented. The primary hypothesis for study 1 is that adolescents’ health and risk behavior displays will have a high positive predictive value (PPV) in identifying behaviors. In our previous work, self-generated alcohol displays were consistently and significantly positively associated with self-reported alcohol behaviors [50,51]. In our previous study, self-reported marijuana users had 1.7 higher odds of displaying marijuana use on social media compared with nonusers. For this study, a target sample size of 400 participants is proposed. A PPV of ≤60% for self-generated risk behavior displays will be considered unacceptably low. Hence, the null hypothesis that the PPV is at most 60% will be tested against the alternative hypothesis that the PPV is greater than 60%. Alcohol use has consistently been the most common risk behavior displayed on social media as well as reported by adolescents in our studies and national surveys. On the basis of the results of our previous study, it is estimated that at least 15% of participants will display alcohol use on social media over the period assessed. With the proposed sample size of 400 participants and an alcohol display rate of approximately 15%, the null hypothesis that the PPV is at most 60% will be rejected with 93% power at the 1-sided .05 significance level, assuming that the true PPV for alcohol display is 80% and an attrition rate of up to 15%. Furthermore, the proposed sample size will provide 55% to 78% power for detecting PPVs for less prevalent risk behavior displays (eg, marijuana use), assuming risk behavior display prevalence rates ranging from 5% to 10%, which is the lower end of prevalence we have seen in studies of older adolescents [52].

The primary goal of study 2 (fMRI) is to examine how the neural correlates of social TDM behavior relate to health and well-being outcomes in a within-participant design. A power analysis was conducted using G*Power based on a prior study that examined whether neural activity in the negative affect network during negative social feedback was related to behavior change (effect size=0.30; *P*=.05; power=0.95). The results indicated that a sample of approximately 110 participants would be large enough to detect a relationship between neural activity and behaviors outside the scanner within participants. In addition, a power analysis aimed at detecting neural differences between 2 time points was conducted based on a study examining reward activity differences within participants between 2-time points (mean of the paired difference 0.09, SD of the paired difference 0.26; *P*=.05; power=0.95). The results indicated that a sample size of 92 would be large enough to detect neural differences within participants. Therefore, a sample of 150 participants should be appropriate for the current design and will compensate for attrition and missing data.

Study 3 will incorporate ethnography and content analysis with data collected monthly. Research staff will immerse themselves in the self-generated content of the participant and take field notes on the participant’s self-generated content using a template comprising the descriptions of the participant’s posts as well as responses to their posts (eg, comments and the participant’s responses to those comments). In addition, notes will be taken on the participant’s overall profile, which may include the content they have been tagged in by others and personal information they use to self-describe. Profile and posts will also be coded for the type and themes of media content presented. Participant content will be assigned to the same research staff each month so that field notes can reflect the context of observable trends and changes within participants over time.

### Social Media Data Collection

#### Platform Selection

We have selected social media platforms based on an affordance approach. Affordances are described as properties of artifacts that can be recognized by users and contribute to their functions or items that present an action possibility [53-55]. The goal of this framework is to increase the likelihood that an assessment or future intervention will hinge on replicable technological qualities as opposed to a platform’s current popularity. This protocol design will focus on platforms with high identity affordances. Identity affordances include key attributes such as the use of individuals’ names and personal photos and support sharing personal content, such as health behaviors [56]. Thus, higher identity affordances are an ideal approach to identify personal disclosures and behaviors. Of the platforms used by adolescents, Instagram, Twitter, and Facebook have been identified in previous studies as high–identity affordance platforms [57,58] and will be the focus of this protocol. TikTok, a newer platform, is widely used by adolescents and has identity affordances that are used to different degrees by adolescents based on whether they post content as an alter ego (eg, cosplay) or themselves. Thus, we will also include this platform for the evaluation of identity-focused content in this protocol.

#### Study Approaches to Social Media Data Collection

Social media data collection will occur over the 2-year enrollment period. This will be data collection through passive observation that requires no ongoing input from participants. With each 6-month check-in, participants will update the study team on their current social media platform use. Given that adolescents typically maintain several platforms and can change them over time, this will allow us to follow these patterns and observe data over the 2-year period. Social media observational data will be integrated into the 3 studies.

In study 1, we will perform a criterion-based content analysis using monthly profile assessments for health behaviors and risks. The main outcomes will be the frequency, prevalence, and content of health and risk behavior displays on social media, and the secondary outcome will be PPV for these displays associated with self-report.

Study 2 will examine social connectedness on social media by examining the number of friends or followers, conversion rates (number of comments/post), applause rate (number of likes/post), and amplification rate (number of shares/post) [59]. In addition, a content analysis will be conducted on peer comments to determine how supportive (ie, positive valence) versus unsupportive (ie, negative valence) peer connections are on social media and the types of support provided, emotional support (eg, expressions of empathy and love), instrumental support (eg, aid and service), informational support (eg, advice and suggestions), and appraisal (eg, information for self-evaluation). The content and network interaction data can be combined to examine the sentiment and support provision, which can even allow for distinctions between message expression and reception [60,61].

Study 3 will incorporate ethnographic field notes collected monthly during the 2-year data collection period. During the monthly observation periods, the research staff will immerse themselves in the self-generated content of the participant for the previous week. The staff will take field notes on the participant’s self-generated content using a template developed for web-based ethnography in our laboratory. The field note template comprises descriptions of the participant’s posts as well as responses to their posts (eg, comments and the participant’s responses to those comments). In addition, notes will be taken on the participant’s overall profile, which may include the content they have been tagged in by others and personal information they use to self-describe. Participant content will be assigned to the same research staff each month so that field notes can reflect observable trends and changes within participants over time. In the field notes template, research staff will reflect on their own interpretations of the participant’s self-generated content as well as any outstanding questions they have about the participant.

### Self-Report Data Collection

The web-based self-report survey measures will be collected at 0, 12, and 24 months. This survey includes measures of demographics, health and risk behaviors, well-being, TDM exposure and use, and parental involvement. These measures will be used across all 3 studies included in this protocol. Full details are reported in Table 1.

**Table 1:** Self-report data collection.

Category	Measure
Well-being measures	RSES^a^ [62,63] UCLA^b^ Loneliness Scale [64] Perceived Stress Scale [65] Warwick Edinburgh Wellbeing Scale [66]
Mental health measures	PHQ-9^c^ [67,68] GAD-7^d^ [69]
Risk behavior measures^e^	Substance use attitudes [70] Substance use social norms [71] Substance use behavioral intentions [72] Substance use current and lifetime [73] Substance use quantity and frequency [74,75]
Health behavior measures	Physical activity [76]
Parent involvement measures	Household media rules [77] Parent-adolescent relationship [78]
Learning disability measures	Diagnosed learning disabilities IEP^f^
TDM^g^ use and experience measures	Emotional responses to social media experiences Preferences for web-based social interactions ADTI^h^ [13,79] Rosen Media and Technology Usage and Attitudes Scale (subscales: Usage and Attitudes) [80]
Demographic measures	Age Race Ethnicity School (grade, public vs private, and web-based vs in person) Birth sex Gender identity Sexual orientation

^a^RSES: Rosenberg Self-Esteem Scale.

^b^UCLA: University of California, Los Angeles.

^c^PHQ-9: Patient Heath Questionnaire-9.

^d^GAD-7: General Anxiety Disorder-7.

^e^Web-based self-report measures were collected at 0, 12, and 24 months. Risk behaviors will examine the use of cigarettes, chewing tobacco, vaping tobacco, cannabis, vaping cannabis, alcohol, cocaine, hallucinogen, opiate, amphetamine, club drugs (methylenedioxy-methamphetamine, ecstasy, and molly), inhalants (nitrous oxide and whippets), and prescription drugs (for nonmedical reasons).

^f^IEP: Individualized Education Program.

^g^TDM: technology and digital media.

^h^ADTI: Adolescents’ Technology Importance and Interactions Scale.

### EMA Data Collection

EMA data will be collected at 0 and 18 months to measure exposure to content about health and risk behaviors. EMA, also called the experience sampling method, is characterized by a collection of data in real-world environments. These assessments focus on an individual’s current or very recent behaviors and are particularly effective for behaviors that occur intermittently [81,82]. EMA avoids the reliance on retrospective reports, which may be subject to biases that challenge their reliability and validity [83]. To measure daily time spent using TDM and content exposure using EMA, surveys will be designed to be brief (<3 min) and allow responses via an SMS text message. Each brief SMS text message survey will begin with the following question: “When you got this text, were you using a digital device?” If participants respond “Y” for yes, these questions will follow: (1) “How long were you on the device in minutes?” (2) “Were you looking at a post or video? (Y or N)” (3) “Please list every topic that was in the post or video (this message is sent with an image that illustrates 9 topics of interest).” To describe their current content exposure, participants can enter all applicable options from the following predetermined content list: E=exercise, A=alcohol, F=food and cuisine, C=cannabis, L=lifestyle and self-improvement, T=tobacco and nicotine, D=drugs, S=shows and entertainment, and O=other. If participants respond “N” to the first prompt, these questions follow: (1) “Have you seen a post or video since we messaged last? (Y or N)” and (2) “Please list every topic from the last post or video you remember” (this message is paired with the topic list image). After completing the survey, participants will receive this message: “Thanks for completing this prompt! Your answers are a contribution to science!”

### Interview Data Collection

We will use a qualitative phenomenological approach to interviews, enabling us to capture individual lived experiences [84]. Our open-ended, semistructured interview method encourages the exploration of concepts important to the participant and provides the researcher with the ability to ask follow-up questions based on the participant’s experience and let their narrative guide the interview process. This creates an opportunity for concepts that might not be detected through other, more structured methods to be introduced [85]. Research staff will conduct in-person or video interviews with participants 6 months after study enrollment and at study conclusion. During these interviews, the research staff will question participants about the social media accounts that they follow, reasons for following the accounts, and how the content from the accounts’ feeds makes them feel. Participants will be encouraged to take out their device and use specific examples from their feed in real time to elicit the nature of each account from their perspective. Our laboratory has piloted similar interviews with adolescents in which study staff reviewed participants’ own social media content in real time, and we have found that adolescents are open with interviewers and eager to teach them about the social media content they see. The data from these interviews (key quotes, observations, and insights) will be analyzed using a constant comparative approach. These findings will be collected and grouped by themes, culminating in a summary that captures key insights, frameworks, and principles to be triangulated with the study team codebook and applied to each participant’s larger list of accounts they are following.

### Neuroimaging Data Collection

#### fMRI Data Acquisition

Brain imaging data will be collected using the 3T magnetic resonance imaging scanner (GE Healthcare MR750) and include longitudinal relaxation time (T1) and transverse relaxation time relaxometry using steady-state sequences (multicomponent Driven Equilibrium Single Pulse Observation of T1 and transverse relaxation time) [86,87]. Structural imaging of anatomical detail for morphometric analyses will be performed using a custom magnetization-prepared rapid acquisition gradient echo sequence, which removes the intensity variations from inhomogeneities in the coil sensitivities. Functional blood-oxygen-level-dependent images may be recorded using a reverse spiral sequence (repetition time=1000 ms, time to echo=32 ms, flip angle=60°, 56 sagittal slices, field-of-view=208 mm, slice thickness=2.5 mm, and voxel size=2.5×2.5×2.5 mm). Each sequence will take 1 to 10 minutes, and the total scan time will be 60 minutes.

#### Collecting Self-Generated TDM Content

Aim 3 of study 1 is to examine whether self-generated versus other-generated TDM content is processed differently and whether neural activity is associated with self-reported health behaviors. To collect self-generated posts, participants will be asked to complete a self-report survey during EMA data collection, which will ask participants to create self-posts that they have or would upload to social media. Participants will upload a picture and create a text post for 25 posts (20 posts will be used in the associated fMRI task). In addition, participants will be asked whether they have already posted the content on social media.

#### Scanning Session

Participants taking part in the fMRI portion of the protocol will complete neuroimaging appointments at 6 and 24 months. Both neuroimaging appointments will consist of 4 neuroimaging tasks aimed at examining the neural processes associated with social connections. Before the scan, participants will be asked to complete a fMRI safety screening form and a series of self-report measures, including measures of well-being and health and risk behaviors. Next, participants will be trained on the fMRI tasks they will be asked to complete during the scanning session. Details regarding the 2 primary functional tasks (ie, self and other messages and Cyberball) are provided in the subsequent paragraphs. Details regarding the 2 secondary functional tasks (ie, peer feedback and social influence) and secondary self-report measures are provided in Multimedia Appendix 1 [70-75,88-107].

#### The Self and Other Message Task

**Figure 2:**
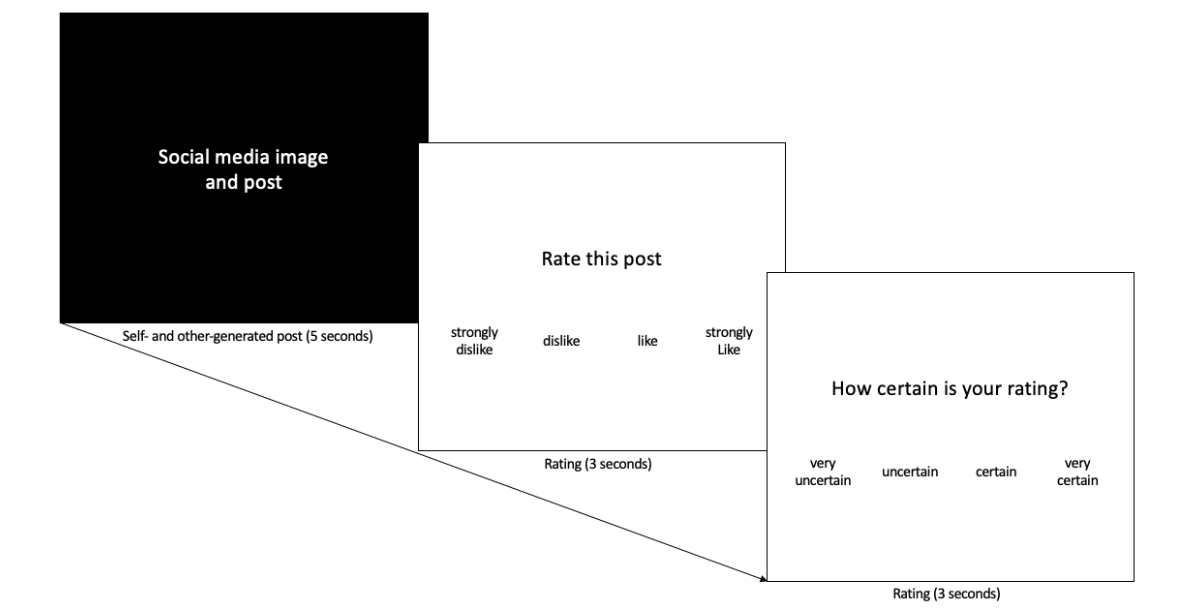
Self and other message task. The self and other message task will measure neural activity during exposure to self-generated and other-generated social media posts.

Participants will first complete the self and other message task as part of study 1 aim 3. Participants will be exposed to 65 social media posts targeting health or risk behaviors. Messages (5 s/message) will be either self-generated using participants’ own data provided in the survey detailed earlier (20 messages) or peer generated (45 messages; categories: vaping tobacco [5], alcohol [5], cannabis [5], exercise [5], nutrition [ie, high-caloric foods; 5], self-image [ie, idealized bodies wearing provocative clothing; 5], civic engagement [5], and generic or neutral [ie, everyday events, such as studying and hanging out with friends; 10]). The other-generated messages will be created by the study team and will be the same stimuli set for each participant. Following each message, participants will answer the question “how would you rate this post on social media” by pressing their pointer finger for 1 star (strongly dislike), middle finger for 2 stars (dislike), ring finger for 3 stars (like), or pinky for 4 stars (strongly like; 3 s). Next, they will rate their preference certainty (on a 4-point scale from 1=“very uncertain” to 4=“very certain;” 3 s)*.* Ratings will be followed by a jittered fixation period (mean 3 s; range 1-5 s), which will serve as an implicit baseline (Figure 2).

#### The Cyberball Task

**Figure 3:**
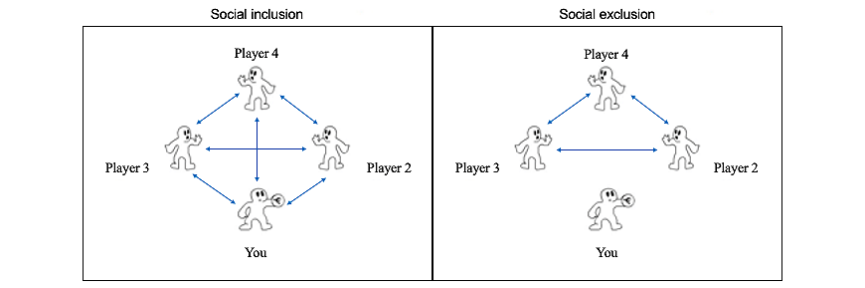
Cyberball task. The Cyberball task is a web-based ball-tossing game that measures neural activity during exposure to social inclusion and social exclusion.

In addition, participants will complete a social inclusion and exclusion task (Cyberball). Cyberball is a game that allows the simulation of both inclusion and exclusion in an fMRI environment [108,109]. In this task, during the first fMRI session, participants will be told that they are engaging in a web-based ball-tossing game with 3 confederate participants playing the game on the web at a remote location. In reality, a preset computer program will control the other 3 players. Before the scan, participants will be shown screenshots of the web-based ball-tossing game and given instructions on how to throw the ball. In addition, they will be told that the only rule for the game is that you can throw the ball to any player; however, you cannot just hold the ball and do nothing. During the first part of the game, the other players will throw to one another and to the participant equally (social inclusion). In the second round, the other players will stop throwing the ball to the participant and only throw to one another (social exclusion). The 2 rounds of Cyberball will each last 178 seconds. The social inclusion round will always be played first, in which all participants will receive the ball equally often. This will be followed in the social exclusion version of the game. The round of social inclusion will always be the first to preserve the psychological experience across participants. These rounds will be preceded by a period in which participants visually track a star as it moves on the screen (105 s). Each round will be separated by a 16-second dot-fixation rest period. This task will always be the final task during the fMRI session. Following the scanning session, participants will complete the Needs Threat Scale to measure self-reported distress following social exclusion in Cyberball [110] and will take part in an interview regarding the study to alleviate any stress experienced from Cyberball. Participants will not be debriefed on the true nature of the Cyberball task after the first scanning session to avoid contaminating the results during the second scanning session (Figure 3).

### Data Analysis

#### Study 1

##### Aim 1

Aim 1 is to determine the frequency and content of self-generated displays of health and risk behaviors on social media profiles. Aim 1a is to test the predictive value of self-generated health and risk behavior content on adolescents’ social media profiles to represent attitudes, social norms, intentions, and behaviors. Hypothesis 1a is that adolescents’ self-generated health and risk behavior content will have a high PPV in identifying behaviors. Aim 1b is to test the moderators of the relationship between self-generated health and risk behavior content and self-reported health and risk behaviors, including parenting involvement and the perceived importance of technology. Hypothesis 1b is that parenting and the importance of technology will serve as moderators such that the associations between self-generated health and risk behavior content on TDM and self-reported health and risk behaviors will be stronger for those with lower parenting involvement and greater technology importance.

In these analyses, the outcome measures will be self-reported behaviors, and predictor variables will be self-generated displays. Measures will be dichotomized based on established threshold values. We will use a generalized linear mixed-effects model with participant-specific random effects and an autoregressive correlation structure to account for repeated measures over time to identify and examine the factors of self-generated displays that predict the corresponding self-reported outcomes. Both univariate and multivariate analyses will be conducted. Study participants’ baseline characteristics, including demographic variables and health and risk behavior variables, will be included as covariates in the multivariate analyses. The least absolute shrinkage and selection operator method for the variable selection of generalized linear mixed-effects models will be used to identify parsimonious models with independent predictors. To test the moderators of the relationship between social media displays and self-reported cognitions (eg, attitudes, social norms, and intentions) and behaviors, we will test interaction effects between social media displays and the moderators of parent involvement and technology importance and include significant moderators in these models. In exploratory analyses, we will examine possible differences in PPV by platform across health and risk behaviors.

##### Aim 2

Aim 2 is to determine adolescents’ real-time TDM exposure to self-generated and other-generated health and risk behavior content using EMA and to test the association between TDM exposure to health and risk behavior content and self-reported health attitudes, social norms, intentions, and behaviors. Hypothesis 2 is that adolescents’ exposure to other-generated health and risk behavior content will have positive associations with attitudes, social norms, and intentions, moderated by technology importance.

The association between TDM content exposure and self-reported intentions and behaviors will be evaluated using a generalized linear mixed-effects model with subject-specific random effects. The moderation effects will be evaluated by conducting a series of univariate and multivariate analyses with the corresponding predictor and median variables, and the Sobel test will be used to formally test for moderation effects [111]. As an additional exploratory analysis, we will determine an accurate measure of adolescents’ daily TDM use. We will not ask participants to attempt to recall a full day or “typical time spent” on TDM because of strong evidence that these measures are inaccurate owing to response and recall bias. Instead, EMA data will be used to calculate daily TDM time as a measure of the quantity of use, which will be reported as descriptive data or incorporated into models across projects as appropriate. First, raw data for the duration of TDM use will be summarized using mean, SD, median, and range from the self-reported data. Second, we will calculate the proportion of time that participants mention that they were using TDM across each participant’s responses of “Y” to indicate that they were actively using TDM. We will use a similar approach to investigate the frequency of content types they are exposed to via TDM. These proportions will be estimated using a generalized linear mixed-effects model with a logit link function and subject-specific random effects. To account for the correlations between time points, an autoregressive correlation structure of order one will be used. Third, we then will use a multilevel modeling approach that captures both within-participant and between-participant variations of the EMA data [112]. This includes subject-specific random effects and an autoregressive correlation structure to account for the correlations within each participant among the repeated measures over time in addition to the covariate of the percentage of time the participant mentions that they were on the web. If the distribution of the time spent is skewed, the data will be log-transformed before conducting the analysis to satisfy the normality assumption.

##### Aim 3

Aim 3 is to use fMRI to examine whether self-generated TDM content versus other-generated TDM content are processed differently and whether neural activity is associated with self-reported health behaviors. Hypothesis 3a is that the ventral medial prefrontal cortex (VMPFC) will show greater activation for self-generated messages compared with other-generated messages. Hypothesis 3b is that sharing more self-generated content on TDM will be associated with increased VMPFC activity compared with sharing more other-generated content. Hypothesis 3c is that VMPFC activity at time 1 will be associated with increased health and risk behaviors reported at time 2.

Data will be modeled at the participant-subject level using the general linear model, as implemented in Statistical Parametric Mapping version 12 (SPM12; Wellcome Department of Cognitive Neurology, Institute of Neurology). Details regarding fMRI data analysis can be found in study 2. The persuasion task will consist of 2 phases: message exposure (5 s), where participants will be exposed to self- versus other-generated messages, and then they will rate the effectiveness of each message (3 s) and provide confidence ratings (3 s), which will be used as baseline ratings for the social influence task. We will model the 5-second period during which participants will be exposed to the persuasive messages as a boxcar. Specifically, we will cross the message source using 2 regressors, self and other, with whether the participant rated the messages as effective. In other words, we will have 2 regressors for each persuasive message condition noted earlier, depending on whether the participant rated the message as effective for that trial, resulting in 4 focal regressors crossing message condition and message effectiveness. Region of interest (ROI) analyses will be performed using independent sample 2-tailed *t* tests and linear regression. The primary ROIs will be regions associated with positive valuations [113], including the VMPFC. The ROIs will be constructed using the association test map in Neurosynth using the search term “valuation.” Individual difference scores will be calculated for the positive valuation network based on a percent signal change score. This score will be created by dividing the average time-series signal (activation) across all voxels within the ROIs during the condition of interest by the baseline condition. The persuasion task will consist of examining differences in neural activity between the following conditions (self-generated>other-generated; self-generated+persuasive>other-generated+persuasive; self-generated+persuasive>self-generated+unpersuasive; other-generated+persuasive>other-generated+unpersuasive). Parallel whole-brain analyses will be performed to determine whether neural regions outside of our a priori ROIs are associated with self-generated versus other-generated content and health behaviors.

#### Study 2

##### Quality Checking

Quality checking of the brain data will be done before and after the preprocessing step to ensure that the results are not driven by abnormalities related to data acquisition or preprocessing (eg, scanner artifacts). All the brain images will be visually inspected for signal dropout or other abnormal data. In addition, motion parameters from SPM12 will be examined, and no runs displaying greater than 3 mm (translation) or 2 degrees (rotation) of head movement during a task run will be used.

##### Preprocessing

Functional data will be preprocessed and analyzed using SPM12. To allow for the stabilization of the blood oxygen level–dependent signal, the first 4 volumes (8 s) of each run will be discarded before analysis. Functional images will be despiked using the 3dDespike program as implemented in the Analysis of Functional NeuroImages toolbox. Next, data will be corrected for differences in the time of slice acquisition using sinc interpolation; the first slice will serve as the reference slice. Data will then be spatially realigned with the first functional image. We then will coregister the functional and structural images using a 2-stage procedure. First, in-plane T1 images will be registered to the mean functional image. Next, high-resolution T1 images will be registered to the in-plane image. After coregistration, high-resolution structural images will be skull stripped using the Computational Anatomy Toolbox 12 for SPM12 and then normalized to the skull-stripped Montreal Neurological Institute template provided by the Oxford Centre for Functional Magnetic Resonance Imaging of the Brain Software Library (“MNI152_T1_1mm_ brain.nii”). Finally, functional images will be smoothed using a Gaussian kernel (8-mm full width at half maximum). Following the preprocessing steps, motion parameters from SPM12 will be examined, and no participants who display greater than 3 mm (translation) or 2 degrees (rotation) of head movement during a task run will be included.

##### Construction of ROI

In both primary fMRI tasks (ie, self and other messages and Cyberball), the primary ROIs will be neural regions associated with social pain [108] and reward processing [114]. ROIs will be constructed using the association test maps in Neurosynth using the search terms “negative affect” and “reward.” Individual difference scores will be calculated for the social pain and reward networks based on a percent signal change score. This score will be created by dividing the average time-series signal (activation) across all voxels within each ROI during the condition of interest by the baseline condition.

#### Primary Task (Cyberball)

##### Statistical Modeling

Data will be modeled at the single-subject level using the general linear model, as implemented in SPM12. The Cyberball (version 5) task consists of 3 phases: social inclusion (178 s), social exclusion (178 s), and visual tracking (105 s), and all 3 phases will be modeled as blocks and convolved with the synthetic hemodynamic response as provided by SPM12. For all tasks, the 6 rigid-body translation and rotation parameters derived from spatial realignment will also be included as nuisance regressors. Data will be high-pass filtered with a cutoff of 128 seconds. Volumes will be weighted according to the inverse of their noise variance using the robust weighted least squares toolbox [115]. The Cyberball task will involve examining neural activity in the contrast (social exclusion>social inclusion).

##### ROI Analyses

To examine neural reactivity to social inclusion and exclusion and its relationship with social media use, well-being, and health behaviors, we will conduct a series of linear regressions and moderation analyses. The specific hypotheses and analyses for each aim are outlined in the following paragraphs.

##### Aim 1

To test whether participants who show greater reactivity in social pain regions during social exclusion will be associated with poorer well-being and risk behaviors at time 1 (hypothesis 1a) and at time 2 (hypothesis 1b), we will test whether social pain activity during social exclusion compared with social inclusion is associated with subjective measures of well-being and risk behaviors at times 1 and 2. Next, to test whether participants who show greater reactivity in reward regions during social inclusion will be associated with better well-being and health behaviors at time 1 (hypothesis 1c) and at time 2 (hypothesis 1d), we will test whether reward activity during social inclusion compared with exclusion is associated with subjective measures of well-being and health behaviors at times 1 and 2.

##### Aim 2

To test whether the relationship between social media use and well-being and health behaviors will depend on the reactivity of participants’ brains to social pain during social exclusion at time 1, we will examine whether social pain activity during social exclusion compared with social inclusion moderates the relationship between social media use (ie, social connectedness measures) and subjective measures of well-being and health and risk behaviors at time 1 (hypothesis 2a) and time 2 (hypothesis 2b). In addition, we will examine whether reward activity during social inclusion compared with social exclusion moderates the relationship between social media use (ie, social connectedness measures) and subjective measures of well-being and health and risk behaviors at time 1 (hypothesis 2c) and time 2 (hypothesis 2d).

##### Aim 3

To test whether changes (time 2–time 1) in reactivity to social inclusion and exclusion will be associated with increased risk behaviors and worse well-being for those who have unsupportive social media experiences over time (hypothesis 3a) and associated with better health behaviors and well-being for those who have a more supportive social media experience over time (hypothesis 3b), we will examine whether changes in social pain activity during social exclusion compared with social inclusion and reward activity during social inclusion compared with social exclusion from time 1 to time 2 moderate the relationship between the social media environment (ie, support measures) and subjective measures of well-being and health and risk behaviors at time 2.

#### Study 3

##### Overview

We will run descriptive statistics and transform skewed outcome variables as necessary to ensure normality. We will address missing data by applying advanced statistical techniques, such as *multiple imputations*, using the multiple imputation procedure in the Statistical Analysis Services and Imputation and Variance Estimation Software Statistical Analysis Services macro. Bivariate analyses will be performed to examine potential covariates and covariates significantly associated with the predictor, and our outcomes will be included in the model.

We will begin by conducting an exploratory factor analysis using principal axis factoring with Promax rotation to examine how social media activities identified through the web-based ethnography load onto latent factors. We will then run a confirmatory factor analysis to establish whether the emerging latent factors are nested into our hypothesized higher-level factor structure, specifically self-generated content and other-generated content. Finally, we will conduct factorial invariance tests using the approach suggested by Kühne [116] to establish whether the same factor structure emerges at all measurement time points and between SGM and non-SGM adolescents.

##### Aim 1

Aim 1 is to test bidirectional relationships between self-generated TDM content and socioemotional well-being. Hypothesis 1a is that TDM engagement with peers (higher vs lower) will be predictive of socioemotional well-being (higher and lower, respectively) at times 1 and 2. Hypothesis 1b is that socioemotional well-being (higher vs lower) will be predictive of TDM engagement with peers (higher and lower, respectively) at times 1 and 2.

##### Aim 2

Aim 2 is to test the bidirectional relationships between other-generated TDM content and socioemotional well-being. Hypothesis 1a is that the consumption of TDM content connected with offline life (higher vs lower) will be predictive of socioemotional well-being (higher and lower, respectively) at times 1 and 2. Hypothesis 1b is that socioemotional well-being at baseline (higher vs lower) will be predictive of the consumption of TDM content connected with offline life (higher and lower, respectively) at times 1 and 2.

To test the direct and indirect associations of self-generated content and other-generated content with socioemotional well-being (aims 1 and 2), we will conduct a cross-lagged panel analysis (ie, a type of structural equation model), with unweighted data and a full information maximum likelihood estimator, using Mplus 8.4 [117]. As illustrated in Figure 1, self-generated content, other-generated content, and socioemotional well-being at *time 0 (baseline)* will be allowed to simultaneously predict these same variables at *time 1* and then again predict these same variables at *time 2*. In cross-lagged models, each person serves as their own control, and results can be interpreted as pertaining to within-person changes; therefore, it is not necessary to include control variables [118]. This analysis will allow us first to investigate the cross-sectional relationships between the variables of interest at *time 0*, *time 1*, and *time 2* (ie, to what extent self-generated and other-generated content are related to well-being), thus adding to prior cross-sectional research. More importantly, it will allow us to examine lagged relationships or relationships between our variables of interest over time (ie, to what extent self-generated and other-generated content at one time point predicts well-being at the next time point, controlling for well-being at the initial time point). These lagged relationships make it possible to longitudinally test whether well-being is (1) an outcome of self-generated or other-generated content (eg, if self-generated and other-generated content at *time 0* predicts well-being at *time 1* and *time 2*), (2) a predictor of self-generated or other-generated content (eg, if well-being at *time 0* predicts self-generated and other-generated content at *time 1* and *time 2*), (3) both (eg, if well-being at *time 0* predicts self-generated and other-generated content at *time 1 and* self-generated and other-generated content at *time 1* predict well-being at *time 2*), or (4) neither (ie, no relationships are statistically significant).

In other words, the lagged analyses will enable us to ascertain the direction of causality between well-being and self-generated and other-generated content on social media. An additional benefit of this statistical approach is the ability to tease out between-person effects (ie, group-level differences) from within-person effects (ie, individual-level differences). Of primary interest to us are within-person effects, which refer to how changes in an individual’s activities affect their well-being over time (and vice versa). Thus, we will be able to investigate whether an adolescent’s social media activities affect their well-being over time and whether an adolescent’s well-being affects the type of social media activities they engage in over time.

In cross-lagged designs, all variables are correlated with each other, resulting in just-identified models. Thus, model fit indices are not informative and do not need to be reported [118]. In addition, further tests of scalar invariance (ie, mean differences across groups in the latent construct) are not appropriate, given that the latent factors at the 3 time points are not independent of each other [119].

From the cross-lagged panel models, all mediated effects of interest will be estimated and tested using bootstrapping. Both the magnitude and statistical significance will be reported, with the significance level set at 2-sided α=.05.

##### Aim 3

Aim 3 is to compare the self- and other-generated TDM experiences of SGM adolescents with those of non-SGM adolescents. This exploratory aim will involve the use of qualitative comparison groups for future hypothesis generation.

### Ethics Approval

This protocol is approved by the University of Wisconsin-Madison institutional review board (2022-1280).

### Data Management

For eligible individuals who decide to participate in the study, all identifying information will be removed from the brain image files, survey, EMA, and interview data, and they will be labeled with a study code before they are transferred for storage or used in data analyses. Only trained research staff will analyze the data. Any forms that contain identifying information will be locked in file drawers or stored in password-protected files and accessible only to this study’s research staff. There may be publications as a result of this study, but no identifying information will be included. Survey data will be collected through the use of REDCap (Research Electronic Data Capture; Vanderbilt University), and fMRI brain images will be collected on the Waisman Center server during the scanning session. Data will be permanently stored on a secure server run by the Department of Pediatrics. Only the study staff, who all have human participants certification, will have access to the data on these servers.

### Reviewer Feedback

Reviewer feedback on the grant and author responses have been provided in Multimedia Appendix 2 [120-122].

## Results

Data collection began in March of 2023 and is ongoing. Our long-term goal is to use the findings from this synergistic protocol program to enhance health messaging campaigns, improve big data surveillance approaches, develop innovative interventions, and advance conceptual models. This protocol will (1) include interdisciplinary studies of TDM use across adolescence using multilevel assessments of neurodevelopment to examine interrelated developmental changes in brain structure and function and complex behavior, (2) examine real-time measures of TDM exposure and use and how this use regulates adolescents’ behavior, and (3) conduct research using converging methodologies to assess the relationship between TDM exposure and sensitive periods in brain development.

## Discussion

### Overview

This protocol will establish a fully integrated interdisciplinary program of research studies that are essential for developing a fundamental understanding of the complex interplay between adolescent health and development and TDM. Previous evidence has illustrated TDM’s connections with adolescent risk behaviors such as increased alcohol behavior and social media exposure, as well as relationships with adolescent well-being such as improved socioemotional health and peer social media connections. The goal of the studies described in this protocol is to address the urgent need to understand how TDM exposure and use affect multiple developmental domains and health outcomes. Study 1 will use TDM to understand the mechanisms behind adolescent health and risk behaviors. Study 2 uses fMRI to understand how positive and negative TDM experiences relate to mental and behavioral health. Study 3 will use a mixed methods design to evaluate self- and other-generated TDM content as predictors of socioemotional well-being in SGM and non-SGM adolescents. Each study uses a 2-year longitudinal design and draws from a shared participant pool. Across studies, observed and measured data will be collected, including observed social media content and fMRI data, self-reported participant experiences and perceptions from surveys and interviews, and EMA for capturing real-time TDM exposure. This protocol prioritizes the dissemination of findings, both to scientific audiences and to the communities across Wisconsin to reach those who participated in this research. Thus, the protocol will enhance the ideas and outcomes obtained through the interactions of the 3 studies. This protocol will provide the scientific approach necessary to advance data-informed theories and conceptual models addressing how TDM exposure and use impact the developmental trajectories and health outcomes of adolescents. Because of the broad potential for advancing research and possible clinical translation of the results, these connected studies portend an opportunity to improve prevention and intervention approaches for adolescent health and TDM.

This project’s data hold exciting potential in four areas:

1. Messaging campaigns: an understanding of key messages regarding health and risk behaviors or socioemotional well-being may inform prevention messaging campaigns. Using data from this protocol, targeted messages (such as tailored advertisements) could be made to automatically
  pop up
  on social media when a specific type of risk-related post is present, providing a link to health education or a web-based intervention.
2. Surveillance: by identifying keywords or phrases associated with health and risk behaviors or well-being, big data approaches could be used to identify or track social media posts focusing on health and risk behaviors or well-being across a larger population or different social media platforms.
3. Intervention approaches: health behavior intention posts may represent an individual’s consideration of behavior change or openness to intervention. Studies support that adults who are considering behavior change have more motivation and willingness to engage in alcohol interventions [123]. Identifying the markers of adolescents’ intention to improve health may allow for novel intervention approaches tailored to this particular stage of development and intentionality.
4. Conceptual models: the findings from this study have the potential to enhance or update conceptual models focused on adolescent technology use, such as the Media Practice Model [124], which supports the idea that adolescents choose to interact with media based on who they are or who they aspire to be. The data from this protocol may also contribute to the development of new models.

### Potential Limitations

A central benchmark of success for our protocol will be our capacity to recruit and retain a shared participant pool of 400 participants for the 3 studies. There are several reasons that support the planning and success of this study. First, authors MAM and ES bring experiences that span projects 1 to 3 in recruiting and retaining adolescent populations. Second, we will pursue recruitment and retention via recruitment ambassadors, who will provide us with a warm and familiar connection with adolescents and families during recruitment. We will leverage both intrinsic motivation, such as promoting emotional connection with the study by providing participants with appealing branded materials, and extrinsic motivation via escalating incentives to retain adolescents. Our investigator team collaboratively designed participant recruitment to occur on a rolling basis over the first 2 years of the protocol period. If recruitment is an issue, we will use alternative approaches, such as targeted social media advertisements and Qualtrics (Qualtrics International Inc) panel–based recruitment, to achieve recruitment goals. If we have retention issues, we will engage our recruitment ambassadors to obtain insights into continued engagement with the participant population. Given that our rolling recruitment efforts will extend over 2 years, we will also have the flexibility to recruit additional participants if early dropouts occur.

A second critical aspect of our study will be to build trust in participants to support their willingness to provide ongoing data in this longitudinal study. Our investigator team will use several evidence-based approaches to optimize adolescents’ trust and comfort in this longitudinal study, including conducting frequent touch points via check-ins every 6 months and providing incentives with every touch point. We will work with trained staff who have centered their research career work on the adolescent population and have training in working with this study population.

### Critical Considerations for Social Media Data Collection

Although adolescents may use other social media platforms, we focus on the selected 4 social media platforms based on several factors. First, authors MAM and ES selected these platforms based on the conceptual approach of affordances, and given this study’s focus on the process of identity development, these 4 platforms represent venues that have strong identity affordances [125,126]. Further, these 4 platforms are popular and used across diverse adolescent demographic groups. Given the length of our planned protocol, there could be a rise of a new popular social media platform or a decline of a planned study platform. If a new platform aligns with promoting identity affordances, we would consider adding it to our study protocol. Content analysis (study 1) and digital ethnography (study 3) procedures require staff training and attention to the coding quality, both tasks that MAM and ES are familiar with owing to past projects. Although big data techniques allow text-based scraping from large data sets, they are limited in that they cannot assess hashtags or all types of photos. Further, big data approaches cannot always ascertain whether content is self-generated or other generated, which are central concepts in this proposal. For this study, we will rely on establishing a *friend* link between our research team profiles and our participants’ profiles. We have several points of evidence to suggest that this approach will work. First, social media users’ desire to accumulate more friends provides an incentive for them to *friend* our research team, as this number is associated with increased social capital [31]. Second, we have used this approach before in several studies to inform friending approaches [127] and collect data [120,128,129]. If a participant wanted to *defriend* us at some point in the study, we would consider this a voluntary withdrawal. On the basis of our previous longitudinal studies, we anticipate that this loss would be <5% of participants.

## Appendix

Multimedia Appendix 1 Secondary functional magnetic resonance imaging data collection.

Multimedia Appendix 2 Peer-review report by the National Institute of Child Health and Human Development Special Emphasis Panel Impact of Technology and Digital Media (TDM) Exposure/Usage on Child and Adolescent Development (P01 Clinical Trial Optional).

## Abbreviations

EMA: ecological momentary assessment
fMRI: functional magnetic resonance imaging
PPV: positive predictive value
REDCap: Research Electronic Data Capture
ROI: region of interest
SES: socioeconomic status
SGM: sexual and gender minority
SPM12: Statistical Parametric Mapping version 12
T1: longitudinal relaxation time
TDM: technology and digital media
VMPFC: ventral medial prefrontal cortex

## Data Availability

Data from this protocol will be deposited into the Eunice Kennedy Shriver National Institute of Child Health and Human Development Data and Specimen Hub repository.
