# Supplementary material for: Effect of Technology and Digital Media Use on Adolescent Health and Development: Protocol for a Multimethod Longitudinal Study
Source: JMIR Res Protoc. 2023 Sep 13;12:e50984. doi: 10.2196/50984 (PMC10534290; doi:10.2196/50984)
Supplement: Multimedia Appendix 1 [file resprot_v12i1e50984_app1.docx]

**Effect of Technology and Digital Media Use on Adolescent Health and Development: Protocol for a Multimethod Longitudinal Study**

Christopher N Cascio, Ellen Selkie, and Megan A Moreno

University of Wisconsin-Madison

## Supplemental Materials

## Secondary neuroimaging data collection

Peer feedback task. As part of secondary data collection participants will complete a peer feedback task during the fMRI appointment. During the peer feedback task, participants will see 60 trials where the stimuli will be 10 of their self-generated messages from the message task. On the top left of the screen, they will be shown the avatar, anonymous username, and their overall ranking of another peer and how they had rated the participant’s pictures. Teens will see ratings from “high” status peers, from “low” status peers, and from “average” status peers for 20 pictures each. High ranking peers were those with an overall ranking of 3.6-4 and depicted with a green border. Average ranking peers were those with an overall ranking of 1.5-3 and depicted with a yellow border. Low ranked peers were those with an overall ranking of 1-1.4 and depicted with an orange border (5 seconds). Participants will see 10 peer ratings that are positive (3 or 4 stars) and 10 peer ratings that are negative (1 or 2 stars) within each of these status categories. Each participant will see 10 of their pictures six times each. While the teen’s picture is on the screen, participants will be instructed to rate how they feel about their peers’ feedback by pressing their pointer finger for very bad, their middle finger for somewhat bad, their ring finger for somewhat good, and their pinky for very good (3 seconds). Ratings will be followed by a jittered fixation period (3 seconds ± 2 seconds) that will serve as an implicit baseline (Figure S1).

Figure S1. Peer feedback task


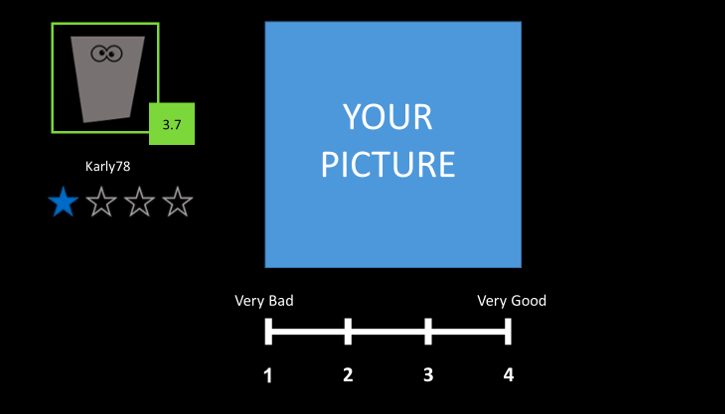


Note: The peer feedback task will measure neural activity to positive and negative peer feedback crossed with peer status.

Social influence task. Participants will also complete an fMRI social influence task. Baseline ratings will be collected during the messages task where participants will rate their preferences of 45 peer-generated posts (categories: vaping tobacco (5), alcohol (5), cannabis (5), exercise (5), nutrition (i.e., high caloric foods; 5), self-image (i.e., idealized bodies wearing provocative clothing; 5), civic engagement (5), and generic/neutral (i.e., everyday events such as studying, hanging out with friends; 10)) based on a 4-point scale from 1=“*strongly dislike*” to 4=“*strongly like*” and preference certainty (4-point scale from 1=”*very uncertain*” to 4=”*very certain*”). The 45 trials will be randomly ordered within participants. Next, during the social influence task participants will complete the second round of the social influence task. Participants will be told that they will be re-rating the same 45 peer posts, however, this time participants would be shown a reminder of how they initially rated the peer posts (2 seconds), then they will be shown information about how other participants in the study rated the stimuli in comparison to the participant (*higher*, *lower*, *same*) (3 seconds). Peer group ratings will be pseudo randomly computer generated to maintain 15 trials (3 seconds each) for each feedback type (*higher*, *lower*, *same*). Finally, participants will be instructed that they will be given an opportunity to update their initial rating if they wished, and to lock in a final response in the scanner (3 seconds). Ratings will be followed by a jittered fixation period (3 seconds ± 2 seconds) that will serve as an implicit baseline (Figure S2).

Figure S2. Social influence task


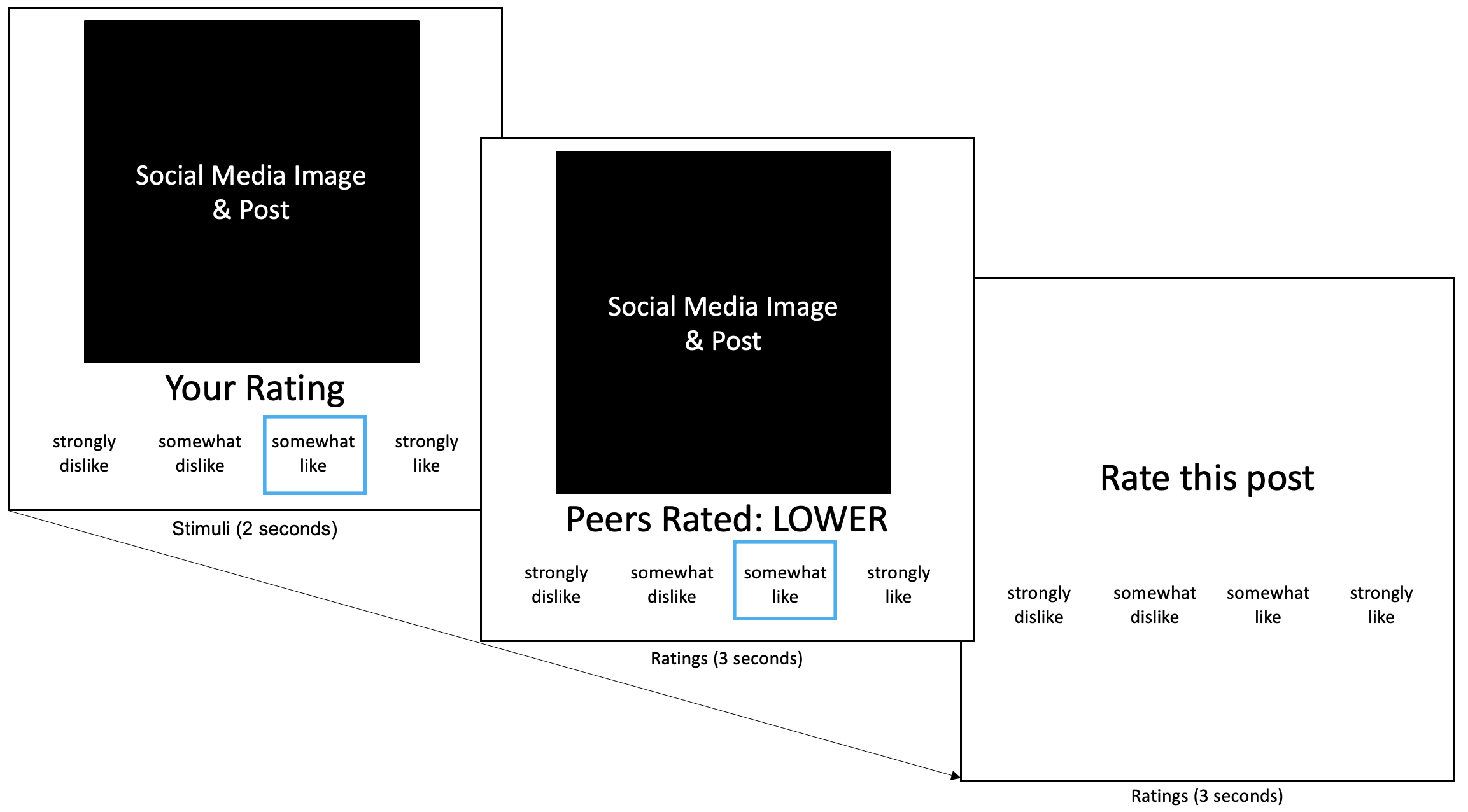


Note: The social influence task will measure neural activity during exposure to social feedback and conformity.

Secondary self-report measures. Before and after the scanning session participants will complete a series of self-report survey measures as part of secondary data analysis questions (Table S2).

Table S2. fMRI self-report measures

| **Category** | **Measure** |
| --- | --- |
|  |  |
| **Risk and Health Behavior Measures** | Exercise and Substance Use Attitudes [70] |
|  | Exercise and Substance Use Social Norms [71] |
|  | Exercise and Substance Use Behavioral Intentions [72] |
|  | Exercise and Substance Use Current/Lifetime [73] |
|  | Exercise and Substance Use Quantity/Frequency [74, 75] |
|  | Exercise and Substance Use Efficacy [88] |
|  | Eating Disorder Examination [89] |
|  | Nutrition [90] |
| **Political Measures** | Social Media Political Engagement [91] |
|  | Political Ideology and Behaviors [91] |
|  | Health and Science Misinformation |
| **Social Media Use Measures** | Bergen Social Networking Addiction Scale [92] |
|  | Problematic Social Media Use [93] |
|  | Perceived Affordances of Instagram & TikTok [94] |
| **Personality Measures** | Narcissism (single item) [95] |
|  | Impulsivity [96] |
|  | Sensation Seeking [97] |
|  | General Life Satisfaction [98] |
|  | Need to Belong [99] |
|  | Optimism [100] |
|  | Hope [101] |
|  | Introversion/Extraversion [102] |
|  | Monitoring Future Milestones [93] |
|  | Health Information Seeking |
|  | Self-Efficacy [103] |
|  | Perceived Social Support [104] |
|  | Revised Life Orientation Test [105] |
|  | Brief Resilience Scale [106] |
| **Demographic Measures** | Socioeconomic Status (Parental Education) [107] |

Note: fMRI self-report measures will be collected in person during the neuroimaging appointments. Substance use behaviors will include vaping tobacco, alcohol, and cannabis use.

## Data analysis

### Peer feedback

Statistical modeling. Data will be modeled at the single subject level using the general linear model as implemented in SPM12. The peer feedback task consists of two phases, peer feedback exposure (5 seconds) where participants will be exposed to positive and negative feedback and peer status which will consist of low, average, and high status. Then participants will rate how they felt about the peer feedback. We will model the 5 second period when participants will be exposed to the peer feedback as a boxcar. Specifically, we will cross peer feedback and self-reported feelings using 6 regressors: peer feedback (positive, negative) with peer status (low, average, high). The primary contrasts of interest will be (positive > negative feedback; positive + low status > negative + low status; positive + high status > negative + high status; positive + average status > negative + average status). Self-reported feelings in response to peer feedback (very bad to very good) will be used in exploratory analyses as a parametric modulator.

Aim 1: To test whether individual differences in reactivity to negative and positive feedback will predict well-being and health and risk behaviors*.* We hypothesize that participants who show greater reactivity in brain regions tracking social pain during negative feedback in the lab will also show poorer well-being and increased risk behaviors at time 1 (6 months; H1a) and at time 2 (24 months; H1b). Participants who show greater reactivity in reward regions during positive feedback will be association with better well-being and health behaviors at time 1 (6 months; H1c) and at time 2 (24 months; H1d).

Aim 2: To test whether individual differences in reactivity to positive and negative feedback moderate the relationship between social media use and health and risk behaviors and well-being. We hypothesize that the relationship between social media use and well-being and health behaviors will depend on participants’ brains’ reactivity to social pain during negative feedback at time 1, such that participants who show greater reactivity in social pain regions during negative feedback will show a more negative relationship between exposure to social media and well-being and health and risk behaviors at time 1 (6 months; H2a) and at time 2 (24 months; H2b). In addition, participants who show greater reactivity in reward regions during positive feedback will show a more positive relationship between exposure to social media and well-being and health and risk behaviors at time 1 (6 months; H2c) and at time 2 (24 months; H2d).

### Social influence

Regions of interest (ROI). In the peer feedback and social influence fMRI tasks the primary ROIs will be regions associated with social pain and reward processing. In addition, the social influence task will examine neural activity in the mentalizing network using the search term “mentalizing”. ROIs will be constructed using the association test maps in NeuroSynth using the search terms, “negative affect” and “reward”. Individual difference scores will be calculated for the social pain and reward networks based on a percent signal change score. This score will be created by dividing the average time series signal (activation) across all voxels within each ROI during the condition of interest by the baseline condition.

Statistical modeling. Data will be modeled at the single subject level using the general linear model as implemented in SPM12. Peer feedback conditions (same, lower, and higher) will be combined with rating outcomes (changed their rating, did not change their rating) as regressors in the model. The initial rating reminder phase (2 seconds), group feedback phase (i.e., peer feedback; 3 seconds), and final rating (3 seconds) will be modeled as separate boxcars. The primary analyses will focus on the group feedback phase for the contrast (group feedback (higher + lower) + changed their rating > group feedback (higher + lower) + did not change their rating). Preference (un)certainty will be used as a parametric modulator.

Aim 1: Examine whether neural processes associated with social influence differ depending on preference (un)certainty with stimuli among adolescents*.* It is hypothesized that increased valuation and mentalizing activity will be associated with conformity when participants indicate greater uncertainty about their own preferences (H1a), whereas increased conflict monitoring activity will be associated with conformity when participants indicate greater certainty about their own preferences (H1b).

Aim 2: Examine whether neural processes associated with social influence moderate the relationship between exposure to behaviors on social media (i.e., frequency) and self-reported behaviors*.* It is hypothesized that increased valuation and mentalizing activity during conformity when participants indicate greater uncertainty about their own preferences will moderate the relationship between exposure to behaviors on social media and self-reported behaviors (H2a). It is hypothesized that increased conflict monitoring activity during conformity when participants indicate greater certainty about their own preferences will moderate the relationship between exposure to behaviors on social media and self-reported behaviors (H2b).
