# Supplementary material for: Effect of Technology and Digital Media Use on Adolescent Health and Development: Protocol for a Multimethod Longitudinal Study
Source: JMIR Res Protoc. 2023 Sep 13;12:e50984. doi: 10.2196/50984 (PMC10534290; doi:10.2196/50984)
Supplement: Multimedia Appendix 2 [file resprot_v12i1e50984_app2.pdf]

SUMMARY STATEMENT
(Privileged Communication)

PROGRAM CONTACT:
JAMES GRIFFIN
(301) 435-2307
griffinj@mail.nih.gov

Release Date: **05/20/2022**
Revised Date:

Application Number: **1 P01 HD109850-01**

Principal Investigator

MORENO, MEGAN A.

Applicant Organization: UNIVERSITY OF WISCONSIN-MADISON

Review Group:	**ZHD1 DSR-Z (50)**<br>**National Institute of Child Health and Human Development Special Emphasis Panel**<br>**Impact of Technology and Digital Media (TDM) Exposure/Usage on Child and Adolescent Development (P01 Clinical Trial Optional)**
Meeting Date:	**04/28/2022**
Council:	**MAY 2022**
Requested Start:	**07/01/2022**
RFA/PA:	**HD22-009**
PCC:	**CDBB -JG**

Project Title:	**A longitudinal study investigating TDM and adolescent health and development: Brain, Behavior and well-Being**
SRG Action:	**Impact Score:42**
Next Steps:	**Visit https://grants.nih.gov/grants/next_steps.htm**
**Human Subjects:**	**30-Human subjects involved - Certified, no SRG concerns**
**Animal Subjects:**	**10-No live vertebrate animals involved for competing appl.**
**Gender:**	**1A-Both genders, scientifically acceptable**
**Minority:**	**1A-Minorities and non-minorities, scientifically acceptable**
**Age:**	**2A-Only Children, scientifically acceptable**

Project Year	Direct Costs Requested	Estimated Total Cost
1	997,419	1,555,233
2	976,419	1,522,489
3	977,433	1,524,070
4	972,433	1,516,274
5	976,433	1,522,511
TOTAL	4,900,137	7,640,577

ADMINISTRATIVE BUDGET NOTE: The budget shown is the requested budget and has not been adjusted to reflect any recommendations made by reviewers. If an award is planned, the costs will be calculated by Institute grants management staff based on the recommendations outlined below in the COMMITTEE BUDGET RECOMMENDATIONS section.

1P01HD109850-01 MORENO, MEGAN

PROTECTION OF HUMAN SUBJECTS: UNACCEPTABLE

RESUME AND SUMMARY OF DISCUSSION: This P01 center application *entitled "A longitudinal study investigating TDM and adolescent health and development: Brain, Behavior, and well-Being"* is submitted in response to RFA-HD-22-009 by the University of Wisconsin with Dr. Megan Moreno as the contact Program Director/Principal Investigator (PD/PI). Technology and Digital Media (TDM) usage and exposure significantly impact adolescent behaviors, well-being, and brain development. The connection between TDM and social media exposure to teenage risk behavior such as substance abuse and well-being or socioemotional health has been previously seen. The overreaching goal of this P01 application is to understand how the TDM exposure and usage affect various adolescent developmental areas and overall health outcomes. The P01 center will collect longitudinal data from diverse adolescent groups, including Sexual and Gender Minorities (SGM), using several resources such as self-report, observed social media data, and functional magnetic resonance imaging (fMRI) and integrate these data to develop conceptual models and new interventions to improve adolescent health. The Program Director is well established and highly experienced in TDM and has established a team of qualified investigators. The application includes three research projects, one recruitment and retention core, and one administrative core. Each component received the following scores:

**Component**	**Score**
Administrative Core	20
Recruitment and Retention Core	25
Project 1- Using TDM to understand mechanisms in adolescent health and risk behavior	38
Project 2- Using functional magnetic resonance imaging to understand how positive and negative TDM experiences relate to mental and behavioral health	55
Project 3- Using mixed methods to evaluate self- and other- generated TDM content as predictors of socioemotional well-being in sexual and gender minority (SGM) and non-SGM adolescents	36
Overall	42

Each score in the above table is the average, multiplied by 10, of all voting committee members' impact scores for each component.

Drs. Megan Moreno and Jens Eickhoff, University of Wisconsin, will lead the **administrative core** for the P01 application. The objective of Administrative Core is to ensure the implementation, integration, and management of all research projects and core activities productively and cost-effectively over the longitudinal design of the projects. The core will provide grant coordination, control, and biostatistical support to Projects 1 and 3 and ensure coordination between research investigators of P01, consultants, and internal and external advisory committees. The core is well-described, with clear and detailed plans to manage all research projects and centers involved in P01. The PI is highly regarded, well-funded in the TDM field, and has strong administrative experience. The Core strategy outlines clear plans for communication between investigators and the coordination of activity by the internal advisory committee that is already in place. However, the role of the external advisory board is unclear. The administrative core includes experienced biostatisticians to support research projects 1 and 3, which is a strength for the application. Overall, reviewers rated the administrative core for the P01 center as outstanding.

The **Recruitment and Retention (R&R) core** is led by Dr. Ellen Silkie, University of Wisconsin, Principal Investigator (PI), and Dr. Megan Moreno, the University of Wisconsin as Co-Investigator (Co-I). The primary objective of the R&R core is to provide the operational structure necessary for planning, implementation, and management of the recruitment and retention of a diverse participant pool over this longitudinal study. The core will ensure efficient, productive, and cost-effective efforts aligned with the overall P01 goal. Overall, the review panel showed a high level of enthusiasm for the core, noting that investigators of the core have shown a high retention rate of adolescents, even in the children and families of low socioeconomic status. The involvement of various recruitment ambassadors from different spheres in aiding other communities and populations for the study is a significant strength of the core. Despite solid enthusiasm for the R&R core, reviewers noted some crucial weaknesses in the core. Panel members raised concerns about the low recruitment of ethical and racial minority youth for the study. Members noted the absence of a community advisory board in the survey and were unclear about the parent role in the study. Overall, the reviewers observed that the strength of this core outweighs weaknesses and scored the R&R core in the outstanding to excellent range.

Project 1, entitled "*Using TDM to understand mechanisms in adolescent health and risk behavior,"* is led by Dr. Megan A Moreno, University of Wisconsin- Madison, as Principal Investigator (PI), and Dr. Dana Litt, University of North Texas Health Center as Co-Investigator. The primary objective of this project is to understand the mechanisms by which Technological Devices and Media (TDM) and social media usage influence health and risk behavior among adolescent youth. This longitudinal study over two years seeks to observe the role of self-generated *vs.* other-generated compelling content on social media towards positive health indicators such as physical fitness, nutrition, and risk behavior such as substance abuse. The study also aims to assess neural mechanisms underlying how self- and other-generated content is processed and affects adolescent behavior using neuroimaging approach. The proposal's strengths include the strong research team and excellent institutional environment to carry out the study. Reviewers observe that though the project asks essential questions about several aspects of TDM use on adolescent development, it misses the opportunity to look at the other critical elements of social media activity, such as direct messaging regarding substance use within social media platforms. Reviewers also raised significant concerns about the study's exploratory nature with few hypotheses. The lack of a track record in recruiting racially and ethnically diverse samples for the analysis is also a significant concern. Overall, the reviewers place the project in the very good to excellent range.

Project 2, entitled *"Using functional magnetic resonance imaging to understand how positive and negative TDM experiences relate to mental and behavioral health,"* led by Dr. Christopher Cascio, the University of Wisconsin as Principal Investigator (PI) in association with Dr. Dhavan Shah, University of Wisconsin, Dr. Eva Telzer, University of North Carolina, Chapel Hill, and Dr. Emily Falk, University of Pennsylvania. The primary focus of this research project is to understand how social media experiences impact the transition of adolescent youth to high school and how social media exclusion and inclusion mediate the relationship between social media engagement and health outcomes. This project seeks to utilize functional magnetic resonance imaging (fMRI) based game called cyberball to examine brain reactivity towards social exclusion/inclusion and its impact on positive or negative health outcomes. The strengths of the project noted by the reviewers include a solid investigative team, a supportive institutional environment, and a novel approach of using social fMRI to measure reactivity to social exclusion and inclusion over time. However, they also noted that the hypothesis is unclear regarding reward reaction to inclusion. It could be an unhealthy dependence on positive reinforcement or simply a relief from exclusion. Despite some novelty in the approach, several key concerns regarding the process and feasibility of the project reduced the reviewers' enthusiasm. Reviewers noted that the test-retest reliability of the cyberball approach had not been established via the pilot study. The lack of

preliminary data or citation of any previous research raises concerns about the feasibility of this integral approach. Reviewers noted that although the PI has the appropriate training in fMRI, inexperience in managing a research study with external funding could be a limitation. Reviewers also raised issues concerning participant confidentiality, data download, and storage. Noting significant weaknesses in approach, the reviewers placed the project in a good to the satisfactory range.

Project 3, entitled *"Using mixed methods to evaluate self-and other- generated TDM content as predictors of socio-emotional wellbeing in sexual and gender minority (SGM) and non-SGM adolescents,"* led by Dr. Ellen Selkie, the University of Wisconsin as Principal Investigator (PI) in association with Co-investigators (Co-I) Drs. Catalina Toma, University of Wisconsin, Julie Lumeng, University of Michigan, and Megan Ranney, Brown University. This research project aims to understand the differential influence of self-and other-generated social media content on the socio-emotional wellbeing of sexual and gender minority (SGM) and non-SGM adolescents. The proposed research examines a bidirectional relationship between engagement with self-and other-generated content on social media and adolescent socioemotional wellbeing across time, using mixed methods such as ethnography and survey research. The reviewers noted numerous strengths in the project, such as a mixed-method approach, focus on the whole marketplace, presence of preliminary data, and a strong investigative team. They were generally enthusiastic about the intense focus on applying social media engagement by SGM youth; however, important concerns were raised about the lack of definitive information about oversampling, recruitment, and retention of SGM youth. Reviewers noted that the applicant did not acknowledge the earlier research that directly observed social media content. The reviewers also raised concerns about how social media data would be archived, coded, and analyzed. In general, the reviewers noted that the application's hypothesis is underdeveloped and more exploratory, rating the project in good to excellent range.

Overall, the panel reviewed the P01 project as having good scientific and technical merit with solid upside potential. The strength of the application lies in the overall goals of the application, exciting questions that allow understanding of behavior, brain activity, health, and wellbeing of adolescent youth in a longitudinal study. The significant sample size of the research and strong focus on the Sexual and Gender Minority (SGM) population is a substantial strength of the application. The strong environment and integration of the research team involving students and new investigators show good synergy for P01 center application. The weaknesses in the individual research projects dimmed the strong overall enthusiasm of the reviewers. Panel members noted that aims are overstated and exploratory to a large extent and lack support of preliminary solid data. They also raised concerns about how content coding is done; there is a lack of specificity and clarity. The panel members appreciate the attempt to utilize the entire social media marketplace in Project 3 but highlight the underutilization of existing research in the field that directly observes social media content. Though panel members noted some concerns about the translatable impact of the study, they appreciate the first start in asking the relevant questions and feel optimistic about the outcome of the study. The reviewers assessed the application to be of medium impact due to concerns about specific projects' approaches and exploratory nature and placed the P01 center application in a very good range.

DESCRIPTION (provided by applicant): This P01 program grant will establish a fully integrated interdisciplinary program of research Projects and Scientific Cores that are essential to develop a fundamental understanding of the complex interplay between adolescent health and development, and technology and digital media (TDM). Previous evidence has illustrated TDM’s connections to adolescent risk behaviors such as increased alcohol behavior and social media exposure, as well as relationships to adolescent well-being such as improved socioemotional health and peer social media connections. The goal of the Projects described in this proposal is to address the urgent need to

understand how TDM exposure and usage affect multiple developmental domains and health outcomes. The three PIs are all located at the University of Wisconsin-Madison, ensuring close collaboration and synergy, in addition to outstanding institutional support and resources, including matching funds. The Projects include Project 1: Using TDM to understand mechanisms in adolescent health and risk behavior. Project 2: Using functional magnetic resonance imaging to understand how positive and negative TDM experiences relate to mental and behavioral health. Project 3: Using mixed methods to evaluate self- and other-generated TDM content as predictors of socioemotional well-being in sexual and gender minority (SGM) and non-SGM adolescents. Each Project utilizes a 2-year longitudinal design and draws from a shared participant pool. Data collection approaches across Projects include observed/measured data including observed social media content and fMRI data, self-reported participant experiences and perceptions via surveys and interviews, and Ecological Momentary Assessment to capture real-time TDM exposures. To support this program, the Administrative Core (Admin Core) will provide organizational and management support to arrange regular meetings across Program collaborators, involve students in the research, leverage biostatistical support and promote dissemination. This P01 proposal will include a Recruitment and Retention Core (R&R Core), supporting a shared participant pool across Projects, and ensuring retention over time. This P01 program will promote synergy in these research efforts through integrated data collection processes over a synergistic longitudinal design, aligned measures and a shared participant pool so that analyses can be structured within and across Projects. This P01 proposal includes a priority on dissemination of findings, both to scientific audiences and to the communities across Wisconsin to reach those who participated in this research. Thus, this P01 program will enhance the scientific knowledge, ideas and outcomes obtained through the interactions of the 3 Projects, the Admin Core, and the R&R Core. This proposed program will provide both the infrastructure support and the scientific approach necessary to advance data-informed theories and conceptual models addressing how TDM exposure and usage impact developmental trajectories and health outcomes of adolescents. Because of the broad potential for advancing research and possible clinical translation of results, these connected Projects portend an opportunity to improve prevention and intervention approaches for adolescent health and TDM.

PUBLIC HEALTH RELEVANCE: Technology and digital media (TDM) have impact on adolescents' behaviors, well-being, and their developing brains. In this P01 program proposal, we will collect data from adolescents over time leveraging several sources, including self-report, observed social media data and functional MRI. This integrated approach to longitudinal data collection from a shared adolescent participant pool will lead to novel analyses and findings, such that this P01 program will advance conceptual models and inform new interventions to improve adolescent health.

OVERALL CENTER, CRITIQUE:

Overall Impact:

The proposed P01, A longitudinal study investigating technology and digital media (TDM) and adolescent health and development: Brain, Behavior, and well-being, provides a rare opportunity for a mixed-methods deep study of 400 teenage participants starting at age 13-15 years old and following them longitudinally for two years to investigate the linkages between technology and media use and brain and health outcomes. The study uses a well-organized shared participant pool to collect a wide range of data on TDM content, health behaviors, risk behaviors, and brain activity. The study is supported by a well-described administrative core that provides organizational and management support, while 400 participants are recruited by a recruitment and retention core. The investigator team (primarily led by Megan Moreno, with other prominent roles by Cascio and Selkie) and institutional

environment are outstanding and well equipped to successfully execute the described projects. In terms of significance, the research about technology and digital media on the development of the adolescent brain and health/risk behavior is extremely important given the vulnerability of the adolescent brain and the ubiquity of screen use among this age group. The novelty of the proposal involves the focus on coding the content of the self-generated and the other-generated content and digital ethnography and merging it with EMA and fMRI data (Projects 1 and 2). Project 3 is also novel and significant in its focus on the socioemotional well-being of a vulnerable population of sexual and gender minority youth. The novel findings of all three projects will be of interest to a wide audience since there is so little research on content of TDM linked with objective outcomes. The primary concern about the study is whether this rich data collection will result in findings that will be able to be translated into the development of interventions or affect clinical practice. For example, it is not clear how the study findings will help with messaging campaigns as described in the proposal. Relatedly, the study design limits the ability to identify causality, and relies heavily on self-reported data for many of the health, behavior, and risk outcomes. More information on data coding and the analysis plan would help improve this limitation. In terms of data collection, there may be a missed opportunity to also focus on screen use duration and timing (especially as it interferes with or delays sleep). A smaller but more addressable concern is that I do not see much detail about racial/ethnic diversity in the proposed participant recruitment plan (although there is effort to recruit sexual and gender minorities). I do not think there will be enough power to make any observations regarding racial/ethnic differences. Overall, this is a rich P01 application with large upward potential for establishing baseline measures of TDM use among teens and some of the associations with brain, behavior, and well-being to make a lasting influence in the field.

1. Significance:
Strengths

- The focus on understanding the relationships between TDM and a range of health and risk behaviors is eminently important – given the pervasive use of screen-based media among teenagers and the potential developmental consequences for health and risk behaviors.
- The focus on adolescence is of great significance during this developmental period when youth begin to engage in more risk behaviors and a vulnerable period. Project 3 focuses on the socioemotional wellbeing of sexual and gender minority youth.
- Screen use is ubiquitous among this age group and given the amount of time spent on the devices the unknown potential for affecting healthy behavioral and brain development is huge.
- The transition to high school is an important development period for social media use and peer influences.

Weaknesses

- Project 1 is unclear in how it plans to use the analyses to get at mechanisms. Thus, it is hard to know how the findings will inform clinical practice or change outcomes among teens.
- It is not clear how the proposed projects will lead to intervention development or messaging campaigns.

2. Investigator(s):
Strengths

- Strong team of researchers, with Dr. Moreno at the helm.

- Most of investigator team based at University of Wisconsin (UW) and has experience working together. If a potential conflict does arrive, there's a sufficient plan for dealing with it through the internal advisory board.
- Very interdisciplinary team with representation from a range of relevant fields: adolescent medicine, neuroscience, emergency medicine, developmental-behavioral pediatrics, communication sciences, applied social psychology, developmental psychology, and clinical psychology
- Biostatistics and informatics expertise is strong
- PI is practicing physician in the UW-Health Adolescent and Young Adult clinic. PI of the social media and Adolescent Health Research Team (SMAHRT). Has served as PI of multiple relevant R grants focused on social media and drug use among adolescents.
- Co-I Dr. Cascio, Ph.D. 2017. Assistant Professor in Communication Studies, School. of Journalism and Mass Communication since 2017. studies why people change their behavior in response to social influence and persuasive messages?

Weaknesses

- No major concerns about the research team.
- Limited mention of the external advisory board.
- Dr. Cascio (Project 2) is junior investigator with less experience and limited external funding.
- Dr. Selkie recently moved to UW Madison, has experience with K23 and R03 grants as sole PI, but no R01 grants or larger.
- How are the internal advisory committee members paid (UW Madison investigators)?
- No external advisory committee identified at this time.

3. Innovation:
Strengths

- Novel opportunity for leveraging multiple types of data collection including content analysis, EMA, survey, and fMRI.
- The use of a shared participant pool organized by the recruitment and retention core creates an innovative opportunity for a broad team of researchers to ask and answer innovative questions and possibly follow up over time.
- The consideration of both risks and benefits of TDM is an important innovation of this proposed research to most prior research in this area.
- Conceptual framework is strong and well-articulated, with multiple theoretical perspectives incorporated (Table 1)
- Focus on identity development and peer connection in context of TDM use is a strength
- Internal Advisory Committee is innovative
- R&R core is innovative
- Recruitment ambassadors is novel in the context of TDM research
- The ethnographic coding of social media use is innovative and important

- Conceptual framework is strong and well-articulated, with multiple theoretical perspectives incorporated (Table 1)

Weaknesses

- May be a missed opportunity to investigate technology duration use, timing especially as it relates to sleep (including sleep onset, duration, and sleep quality).

4. Approach:

Strengths

- Shared participant pool to look at a range of outcomes across three projects efficient and effective. Project 1 focuses on behavior, Project 2 on neuroscience, and Project 3 on socioemotional well-being of sexual and gender minorities. This data collection design is cost efficient and allows for rich data analysis.
- R&R core plus the admin core will allow for opportunities for other researchers to conduct auxiliary analyses with the rich data.
- Timelines are provided for data collection in the first two years of the study and time for additional analyses during the proposed period of award. Secondary data analysis (or additional studies) could be continued after the study period ends.
- Two fMRI studies per participant allows for rich objective outcome measures in sample of 150 participants.
- Resource and data sharing plans are well-described and strong.
- Brain, behavior, and well-being overarching plan is operationalized well in the approach
- Focus on both creation and consumption of TDM by adolescents is a strength, program project takes a “whole marketplace” approach
- Conceptualization of levels of influence, mediators and moderators described in the project research plans are a strength, align well with conceptual framework (integrated framework and research/analysis plans)
- EMA of social media content exposures and quantity of use – twice/day for 5 days.

Weaknesses

- Self-reported data for many of the behavior and risk measures – although many scales are validated, the participants are young and may not be reliable reporters of many of the outcomes. I would prefer more objective assessment or direct observation of outcomes when possible.
- Not clear if there will be investigation into gender or racial/ethnic differences in effects of TDM.
- Moderation appears to focus on rather vague constructs of technology importance and parenting without well-developed hypotheses.
- Recruitment will require that participants will have at least one social media account among the four that have been selected. Will there be a prioritization of participants who are active on 3-4 platforms as opposed to just 1-2? Will this result in selection bias toward heavier TDM users? At a minimum there will be a selection bias against anyone who barely or rarely uses social media as they may not be eligible to participate.
- Unclear methods of recruiting a diverse sample (will use recruitment ambassadors). No pilot work was described to demonstrate the capacity to recruit participants from underrepresented groups. Will overrecruit 30 gender minority youth and 50 sexual minority youth, but purpose of

this over-recruitment was unclear. Also, they are recruited from a clinic; are they representative of gender and sexual minority youth in general?

- While there is information about recruitment of sexual and gender minorities, will this affect the other participant sample population that affects other aims?
- While the focus on content is important and novel, there may be a missed opportunity to investigate technology duration use, timing especially as it relates to sleep (including sleep onset, duration, and sleep quality).
- It is unclear if 150 participants be sufficient for the neuroimaging analyses to identify differential susceptibility. More description of how fMRI will be used, and how data integrated with social-behavioral data would've been helpful (minor)
- n=150 participants (Group A—from a total of 400 participants) will complete two scanning sessions over a 2-year period as part of Projects 1 and 2. No evidence is provided that this sample size is large enough to identify typical and atypical trajectories associated with TDM usage.
- Very exploratory and descriptive aims in general, i.e., do latent class analysis (LCA) predict social, brain, and behavioral outcomes? Longitudinal LCA analyses will be conducted with only two time points. What is the evidence that there are different types/groups?

5. Environment:
Strengths

- University of Wisconsin environment is excellent with institutional and collaborative support to support the probability of success of the proposal.
- Physical resources are available to the investigators are more than adequate.
- Matching funds are provided by the University will benefit the project.

Weaknesses

- No concerns about the institutional environment.

TDM P01 as an Integrated Effort

- The application describes coordination, cohesiveness, and synergy across the research projects and core.
- The projects benefit from each other by using the shared participant pool for rich data collection and thus the investment of each project goes further than when conducted in isolation.
- The matching funds from the University of Wisconsin are also beneficial to the overall success of the proposal.

Protections for Human Subjects

Adequate

- Acceptable Risks and/or Adequate Protections
- No concerns about human subjects' risks.

Data and Safety Monitoring Plan (Applicable for Clinical Trials Only):

- Not Applicable (No Clinical Trials)

Inclusion of Women, Minorities, and Individual Across the Lifespan

- Sex/Gender: Distribution justified scientifically
- Race/Ethnicity: Distribution justified scientifically
- For NIH-Defined Phase III trials, Plans for valid design and analysis: Not applicable
- Inclusion/Exclusion of Children under 18: Including ages <18; justified scientifically
- Scientifically justified diverse distribution, although it is not clear there will be enough minorities in the population to make observations regarding race/ethnic differences. Necessary inclusion of <18-year-olds due to interest in TDM during this age range (enrollment at age 13-15). More information could be provided about the expected distribution of minorities in the participant pool and how they will recruit to ensure such diversity. They will use recruitment ambassadors to recruit sexual and gender minorities. I do not see effort about recruitment of racial/ethnic minorities.

Vertebrate Animals

- Not Applicable (No Vertebrate Animals)

Biohazards

- Not Applicable (No Biohazards)

Resource Sharing Plans

- Acceptable
- No concerns about resource sharing plan. Detailed plan is well described, and Dr. Moreno has experience in participating in a data consortium.

Budget and Period of Support

- Recommend as Requested

ADMINISTRATIVE CORE:
Principal Investigator: Dr. Megan Moreno
Overall Impact Score: 20

(Description as provided by applicant): This proposed P01 program has the goal of creating a locus of research examining the pathways by which technology and digital media (TDM) exposure and usage impact developmental trajectories and health outcomesin adolescence. The Administrative Core (Admin Core) of this proposal will provide the necessary support to the program investigators, staff and collaborators over the longitudinal design of the proposed Projects. This Core will serve as the common channel through which all scientific, administrative and fiscal communication occur tofacilitate the exchange of information between Project investigators and staff internally, as well as disseminatingthe findings of the Projects and communicating research advances to the public. The Admin Core will thus provide coordination and an organizational backbone for the 3 Projects and 2 Cores involved in this P01 proposal. The Admin Core of this P01 program proposal will focus on five Specific Aims. First, this Core will provide grant coordination, organization and management services. Second, the Admin Core

will organize and coordinate biostatistical support for Projects 1 and 3. Investigators will also identify novel analysis ideas based on early findings towards triangulating data across all 3 Projects given the shared participant pool. Third, the Admin Core will ensure ongoing involvement of this P01 proposal's team of co-investigators, consultants, and the internal and external advisory committees. Given that the three PIs are all located at the University of Wisconsin-Madison, this Program proposal includes an Internal Advisory Committee spanning diverse disciplines across areas of technology and digital media, as well as adolescent health and development. This P01 proposal includes strong institutional support including matching funds from the involved departments and schools, and main campus at UW Madison. Fourth, as a university-based research team, all Program investigators are committed to the ongoing growth of the next generation of researchers. Thus, a specific aim ofthis Core will focus on engaging diverse undergraduate and graduate students in all 3 Projects. Finally, the fifthaim will focus on ensuring timely and targeted dissemination across local, national and scientific and community audiences. This Admin Core team includes Core Lead and P01 PI Dr. Megan Moreno, the biostatistical team includes Dr. Jens Eickhoff whom Dr. Moreno has worked with across several previous projects, the Admin Coreadministrative and research staff who all have experience and existing collaborations with Dr. Moreno, and Communication Lead staff member Ms. Bushman who will ensure that our learnings and findings reach communities to optimize the impact of this collaborative interdisciplinary research program.

ADMINISTRATIVE CORE, CRITIQUE 1:
Strengths

- The Administrative Core will provide necessary organizational and management support for regular meetings across collaborators (including internal and external advisory boards) as well as biostatistical (for Projects 1 and 3) and dissemination support. In addition, the Admin Core will help with coordinating student experiences.
- Dr. Moreno (PI) has extensive and continuous funding as the PI on topics related to TDM and well-being. Moreno also has administrative experience serving as Vice Chair of Academic Affairs at University of Wisconsin.
- Administrative core will set the timeline, calendaring as well as providing the meeting agenda and minutes.
- Administrative core will also handle fiscal communication.
- Presumably regular meetings will be used to help coordinate the use of shared recruitment, data collection, and measures across the 3 projects.
- All three PIs are based at the same site, University of Wisconsin- Madison makes management of the study straightforward. Administrative Core personnel include Dr. Jens Eickhoff (Senior Scientist/Biostatistician) and Ms. Bushman as communications lead specialist. There are other staff involved in Admin Core not named.
- Risk mitigation plans are well described for the participant sharing plan, the personal protective gear, and management of multiple PIs.

Weaknesses

- Use of an External Advisory Board is mentioned but not well described.
- It is not clear why Project 2 does not need statistical support.

ADMINISTRATIVE CORE, CRITIQUE 2:
Strengths

- PI Dr. Moreno has been conducting research in this area as PI of several NIH and NIJ funded projects
- PI Dr. Moreno runs a current research program called the social media and Adolescent Health Research Team (SMAHRT) with ongoing funding and research productivity for 13 years
- PI Dr. Moreno also led the inception of a foundation-funded program on technology and adolescents that has ongoing external advisory boards of adult experts and of youth experts; they made grants to six projects
- Experienced biostatisticians will operate out of this Core
- A detailed meeting schedule for management of the P01 investigators and their teams is included
- An internal advisory committee has been formed
- An external advisory committee was not formed for the proposal, but the plan to recruit members if awarded is included. Given their experience with the ongoing external advisory boards of other projects, this plan seems acceptable.
- They include a list of benchmarks for success which will be their evaluation of the program
- There is a cost-sharing part of this P01 where the university will contribute $500K to fund a postdoctoral fellow

Weaknesses

- None Noted

RECRUITMENT & RETENTION CORE
Principal Investigator: Dr. Ellen Marie Selkie
Overall Impact Score: 25

(Description as provided by applicant): This proposed P01 program has the goal of creating a locus of research examining the pathways by which technology and digital media (TDM) exposure and usage impact developmental trajectories and health outcomes in adolescence. The Recruitment and Retention Core (R&R Core) of this proposal will provide the necessary support to our shared pool of participants who will contribute across all 3 Projects. The R&R Core will serve as the common channel through which all support and resources for participants occurs to ensure a successful recruitment and consistent levels of participant engagement over this longitudinal study. The R&R Core will thus provide coordination and an organizational backbone for the participant experience in this P01 program. The R&R Core of this P01 program will focus on four Specific Aims. First, the R&R Core will leverage our Recruitment Ambassadors and consultant towards recruiting a diverse group of adolescent participants as our shared participant pool. Rolling recruitment will occur over years 1 and 2 of this P01 program to achieve a shared participant pool of 400 adolescents. Adolescents ages 13-15 years at enrollment will be recruited across different settings for a 2-year research study experience. We have worked with our Recruitment Ambassadors to create an initial list of recruitment opportunities that would be refined and implemented at program inception. Second, the investigators and staff will complete initial and ongoing regulatory processes including human subjects' protocols. Third, the R&R Core will organize and

communicate timelines relevant to each participant's entry time point over our two-year rolling recruitment. Each participant will be scheduled for every 6-month data collection experiences and assigned to a group (Group A: n=150 participants with fMRI, Group B: n=250 participants without fMRI). And finally, the fourth aim of the R&R Core will be to retain participants for the 2-year study. We have incorporated the perspectives of Dr. Moreno's research team's Youth Advisory Board (YAB) into our proposed retention plans, which include incentives that incorporate internal and external motivations for serving as a research participant. We are excited about our dynamic R&R Core team, including Core Lead Dr. Selkie who brings experience recruiting and retaining early adolescents, and P01 PI Dr. Megan Moreno who brings experience retaining college students in a 5-year study. Research staff on this Core include Victoria Adkins, who has worked with Dr. Selkie the past 4 years on a longitudinal study of early adolescents. Our Recruitment Ambassadors will be valued partners in our efforts on this R&R Core. This P01 program provides the opportunity for alignment in our research efforts through a shared participant pool that all 3 Projects will draw from, and synergy by triangulation of participant data across Projects. Thus, this Core will enhance the scientific knowledge, ideas and outcomes obtained through the interactions of the R&R Core, the Admin Core and the 3 Projects.

RECRUITMENT & RETENTION CORE, CRITIQUE 1:

Overall Impact:

The recruitment and retention core of this project is essentially a comprehensive description of the recruitment and retention practices of the project as a whole. In this regard, the recruitment and retention core holds promise to be a very solid vehicle for recruitment of a diverse and representative cohort of Wisconsin adolescents. The investigators of the core have a demonstrated very high retention rate in this age group, even in children and families of low socioeconomic status. Strengths of the approach of the core entail its recruitment ambassadors, which are composed of key players in different spheres of potential recruitment and including physician ambassadors who can oversee recruitment from clinics, including an LGBT clinic as well as community centers advocacy groups, and schools. The diverse sources of recruitment are likely to yield robust results, in addition to the contingency plan of using social media itself as a recruitment tool. In addition, the core is tasked with maintaining IRB approvals of linked protocols and consents. The feasibility of this approach is suggested by the positive and experienced interactions of the PIs with the local IRB that has special expertise in social media related proposals. Other strengths of the recruitment include input from youth focus group itself as well as issuance of logo-embossed gifts. In general, the proposal includes best practices for retention and incentivization with both intrinsic and extrinsic financial motivation. In addition, the base camp progress report monitoring software has been successfully used by the team and holds potential to identify potential flagging recruitment for correction. One notable omission of input was a community advisory board (including parents of participants) that could comment on planned and ongoing recruitment and procedures for a longitudinal study. However, this is only a minor concern considering the shorter duration and representation of ambassadors. Enthusiasm for this core was very high.

Strengths

- The investigators of the core have a demonstrated very high retention rate in this age group, even in children and families of low socioeconomic status.
- Recruitment ambassadors are key individuals who can oversee different spheres of potential recruitment, to capture kids from all kinds of contexts. These include physician ambassadors who can oversee recruitment from clinics, (including an LGBT clinic) as well as community centers, advocacy groups, and schools.

- Positive and experienced interactions of the PIs with the local IRB that has special expertise in social media related proposals
- Recruitment designed with input from a teen focus group
- Recruitment features “swag” with study logo, for more emotional investment in participation by teems
- Base camp progress report monitoring software has been successfully used by the team, and holds potential to identify potential flagging recruitment for correction

Weaknesses

- No external advisory board, such as with community stakeholders or even parents of participants, who might comment on current social media fads, apps, or unusual concerns that might prompt additional probes or assessments or controls.

RECRUITMENT & RETENTION CORE, CRITIQUE 2:

Strengths

- The core lead (Selkie) has substantial experience recruiting adolescents into research, including TDM related research.
- Plans for intrinsic and extrinsic motivator incentives is sound
- The R&R core is novel and has high utility is serving to create a pipeline of participants for research projects and to engage the community in the P01
- Services of the R&R core are well described and are integral to the overall program project
- Dr. Selkie’s prior research and recruitment under her K award are a strong qualification

Weaknesses

- Dr. Selkie does not have a long-track record of experience in research, especially at the scale of this P01, overall (minor)
- There is no community advisory board (minor), should be added
- It is unclear if recruitment will be successful in achieving a racially/ethnically diverse sample (minor)

PROJECT 1: USING TDM TO UNDERSTAND MECHANISMS IN ADOLESCENT HEALTH AND RISK BEHAVIOR.
Principal Investigator: Dr. Megan Moreno
Overall Impact Score: 38

(Description as provided by applicant): Adolescence is a critical time for the development of health behaviors such as physical activity and risk behaviors such as alcohol use. One-way adolescents learn about and model their behaviors is via technology and digital media (TDM), particularly social media. Adolescent behavior can be influenced by content that adolescents display (self-generated) on social media, as well as content they consume (other-generated). The long-term goal of this research is to understand mechanisms by which social media influence health and risk behavior towards the development of interventions to promote healthy behavior and reduce risk behavior. The objective of this Project 1 is to understand patterns and content of displayed health and risk behavior on social media that is created by, and consumed by, adolescents. We will determine connections between three key concepts through our Specific Aims, including: 1) what adolescents display on TDM about their own health and risk behaviors via self-generated content, 2) the displayed TDM content adolescents are

exposed to in real-time about health and risk behaviors, and 3) adolescents' self-reported health and risk behavior attitudes, social norms intentions and actions. Our third aim will assess neural processes underlying how created and consumed TDM content is processed and associated with health and risk behavior. The PI, Co-I and consultant on this Project bring over a decade of work in the area of adolescent behaviors displayed on social media, including several NIH-funded studies in this area. Our preliminary data indicates that for older adolescents, self-generated risk behavior content on TDM closely aligns with self-reported behavior, and that Ecological Momentary Assessment (EMA) is feasible to use with adolescent populations to assess real-time TDM use and content exposure. This study will use a longitudinal study design, collecting data over 2 years. The shared participant pool for this P01 program will be leveraged to recruit the sample of 400 adolescents aged 13-15 years at enrollment. Data collection approaches will include social media observation of health and risk behaviors, including self-generated and other-generated. This social media observational data will be linked to self-report survey data including attitudes, social norms, intentions, and behaviors. Moderators will include technology importance and parent involvement. Data collection will also include Ecological Momentary Assessment (EMA) to determine adolescents' exposure to self-generated and other-generated content across TDM in real-time. Finally, investigators will utilize social media data during a fMRI scan to determine whether self-generated and other-generated content is processed differently. This project aligns with the current RFA as utilizes multi-level assessments of health and development, including real-time measures of TDM exposure.

PROJECT 1, CRITIQUE 1:

Significance: 3
Investigator(s): 2
Innovation: 3
Approach: 5
Environment: 2

Overall Impact:

Project 1 seeks to investigate the links between TDM and health and risk behaviors. The strategy involves doing behavior-related content analysis over the first two years 2 years and linking it with a range of scales of well-being (self-esteem, loneliness, perceived stress, strengths and difficulties, Warwick well-being), physical health behaviors (nutrition, physical health), mental health (depression, anxiety), and risk behaviors (substance use). EMA data and fMRI data will also be collected at two time points for each participant. While the focus on content is significant and novel, and the combination of multiple measurement techniques will allow for a large amount of testing, I am concerned about the ability of this study to identify mechanisms that can inform intervention or clinical research. I also have concerns about the ability to standardize coding for content analysis (given the use of humor/irony/inside jokes) and whether the 4 social media platforms are the right selection of platforms. More information about how the content coding will occur and the analysis plan will help inform the extent to which this data collection will have a strong impact. At present the data collection is very strong, but analyses are more exploratory with few hypotheses.

1. Significance:
Strengths

- This project focuses on an important and underexplored topic – what are the mechanisms in explaining linkages between TDM and a range of health, well-being, and risk behavior

outcomes. The stakes are high given the pervasive use of screen-based media among teenagers and the potential developmental consequences for health and risk behaviors.

- The goal is admirable – to develop feasible, scalable, and tailored interventions to reduce health risk behaviors.

Weaknesses

- What are the hypothesized mechanisms that will be tested? It looks more like it is a test of associations than a test of mechanisms. Without having clear mechanisms to test, it is not clear how the research will be able to directly inform intervention development or clinical treatments.
- Proposed research questions are exploratory and are not hypothesis driven.

2. Investigator(s):
Strengths

- Dr. Moreno (PI) will work closely with Dr. Litt, Dr. Pagoto, Dr. Selkie, and Dr. Cascio who bring appropriate and excellent training and publication records. All have been recognized with various awards in their field.
- Dr. Moreno (PI) brings previous pilot experience with her recent R01 showing the role of self-generated social media content and alcohol consumption, which is right in line with this project, prior EMA studies,
- Research Specialists/Assistants Anna Joliff and Reese Hyzer are already identified and bring both experience and expertise.
- Most of investigator team based at UW and has experience working together. If a potential conflict does arrive, there's a sufficient plan for dealing with it through the internal advisory board.

Weaknesses

- Dr. Cascio is a newer investigator.

3. Innovation:
Strengths

- Novel combination of EMA, social media content, collection of outcomes of risk behavior, and fMRI.
- Using LCA to identify differential susceptibility is novel and important
- Prior work on TDM and health behaviors has primarily focused on college age population. This study focuses on novel population of adolescents.
- Measurement of TDM incorporates quality measures in addition to quantity measures as previously investigated.
- Scales and survey metrics are well-justified and based on scientifically justified ones.

Weaknesses

- Focus of risk behavior primarily on substance use/abuse feels narrow. Have the researchers thought to include other risk behaviors including sexual activity, reckless driving, gun use, violence, self-harm, etc.?
- Are the social media platforms – Facebook, Twitter, Instagram, and TikTok the most current and relevant ones to this age group (13–15-year-olds)? This needs to be reevaluated at the

beginning of the study and more regularly throughout the study. The social media landscape is always evolving, and the proposed platforms explored in this study may not be current and thus the proposal may not be studying the most novel forms of social media.

4. Approach:

Strengths

- Study design incorporates multiple types of data collection. Social media content analysis will occur every month, over the duration of the first 2 years. 5-day EMA data collection will occur at the study inception and 18 months later. Neuroimaging data will occur at two time periods per participant. And finally, self-report will occur at baseline, year 1 and year 2. This rich data collection allows for extensive data mining and hypothesis testing.
- Aim 1 seeks to study the frequency and content of self-generated content and the links with a range of relevant health and risk behaviors.
- Aim 2 seeks to understand exposure to other generated content which will be collected through a series of short questions asked through EMA.
- Aim 3 seeks to incorporate fMRI to test whether prefrontal cortical activity is greater among self-generated content than other generated content.
- Data collection and analysis is well timed across 5 years of the project timeline.

Weaknesses

- Even though criterion-based content analysis will have codebooks, is it possible to standardize across all participants, especially when content is often mixed with irony, humor, inside jokes, and variable meanings across people.
- More information is necessary about what mechanisms are being tested and how learning about the mechanisms will help inform intervention development or clinical practice.
- More information is necessary about the analytic plan and the attempt to get at causality/mechanisms rather than simply associations.
- Self-reported data on health outcomes for both physical activity and nutrition are prone to error. Direct observation through actigraphy for both physical activity and sleep could be used for better assessment of movement.
- What is meant by technology importance and parental involvement as moderators? More justification is needed to understand what the hypotheses are. And if these measures are being treated as moderators they are not being tested as mechanisms.
- Statistical analysis for Aim 2 describes using the Sobel test for moderation, but Sobel test is used for mediation analyses.
- Household media engagement survey does not appear to be validated but rather based on agreement with statements related to the AAP recommendations.
- What do the profiles of the research team look like when an adolescent friends them? Will it be a younger member of the research team who may appear to be in a peer group, or will it look like a possible relative or older friend? This may affect willingness for individuals to accept. (Not much social capital to be gained for adding grandpa your social media account, for example.)
- TikTok is described as a newer social media platform. While this is true, it is still about 5-6 years old. Some of the platforms may not be relevant to the participants by the time this study begins. I am concerned that the list of platforms may need to be revised.

5. Environment:

Strengths

- University of Wisconsin, Madison environment is excellent with institutional and collaborative support.
- Physical resources are available to the investigators are more than adequate.
- Matching funds are provided by the University.

Weaknesses

- No concerns about the Environment.

Protections for Human Subjects

- Acceptable Risks and/or Adequate Protections
- Incentives seem fair and non-coercive ($30-$100 per portion)
- Nice for teens to get a printed photo of their brain as an incentive for participating in fMRI study, may have positive consequences for them thinking about their brain health

Data and Safety Monitoring Plan (Applicable for Clinical Trials Only):

- Not Applicable (No Clinical Trials)

Inclusion of Women, Minorities, and Individuals Across the Lifespan

- Sex/Gender: Distribution justified scientifically
- Race/Ethnicity: Distribution justified scientifically
- For NIH-Defined Phase III trials, Plans for valid design and analysis: Not applicable
- Inclusion/Exclusion of Children under 18: Including ages <18; justified scientifically
- Study is designed to include males/females and a distribution of race/ethnic backgrounds to match the general population however, I am concerned that due to smaller sample sizes of minorities there will not be enough power to make any observations regarding racial/ethnic differences. Due to the interest in adolescent TDM use, the study requires enrollment of <18-year-olds.

Vertebrate Animals

- Not Applicable (No Vertebrate Animals)

Biohazards

- Not Applicable (No Biohazards)

Resource Sharing Plans

- Acceptable
- No concerns about resource sharing plan. Detailed plan is well described, and Dr. Moreno has experience in participating in a data consortium

Budget and Period of Support

Recommend as Requested

- P01 proposes $500,000 in matching funds from UW to help with additional costs of the program, specifically in the form of a post-doc.

PROJECT 1, CRITIQUE 2:

Significance: 1
Investigator(s): 3
Innovation: 4
Approach: 5
Environment: 1

Overall Impact:

Project 1 seeks to assess adolescents' TDM (specifically social media use, captured by "friending"/connecting to each of the adolescent's profiles using a study account) to identify mechanisms affecting adolescent health and risk behavior, including the role of self-generated vs. other-generated persuasive content on both positive health behaviors like nutrition and physical activity as well as risky behaviors like drug use. The study design is longitudinal and observational over 2 years and includes (aim 1) content analysis of adolescents' usage on 4 social media platforms related to health and risk behaviors and whether self-generated images predict these self-reported behaviors; (aim 2) EMA to capture real-time reports of social media usage and health/risk behaviors at baseline and 2-years later and the association with attitudes, social norms, intentions, and behaviors; and (aim 3) a neuroimaging study on a subset of 150 participants to examine reaction to self- vs. other-generated persuasive content using the adolescent's own images. Strengths include a strong investigative team with preliminary data and sufficient facilities/resources, as well as innovation especially in aim 3 using adolescents' own self-generated images and comparing to other-generated images, as well as the focus on not only risky behaviors but also positive health behaviors. The key weaknesses include that many interactions (e.g. direct messages within social media platforms) are not captured even though literature indicates this is where a lot of communication regarding risk behaviors occurs, and there are available tools/apps like EARS to unobtrusively collect and archive all of these data; weak assessments of diet (1 dietary recall at each timepoint) and physical activity (3-item self-report), the lack of an overall conceptual model to link the theoretical constructs with the anticipated associations/hypotheses, a relatively homogenous sample in terms of race/ethnicity, and some clarity needed on the methods for Aim 3. The identified weaknesses are addressable if the team will seek to diversity the sample and consider ways to address the weaknesses in the approach.

1. Significance:

Strengths

- The application articulately and convincingly argues of the significance of studying risk behaviors and health behaviors during adolescence as it relates to TDM use and brain development.
- The inclusion of parental involvement as a moderator (e.g., household rules) is important given it is described in the American Association of Pediatrics guidelines, yet perhaps less so for these mid/older adolescents as compared to younger children.

- The focus on self- vs. other-generated persuasive content is significant given the ubiquity of content generation/exposure in adolescents' lives and nearly half of adolescents reporting they are online "almost constantly" with a lot of that time spent on social media.

Weaknesses

- None noted.

2. Investigator(s):

Strengths

- This is a tremendously well qualified team including a PI who has NIAAA-funding demonstrating an association among young adults' self-generated alcohol posts and self-reported drinking, an R01 that has published 24 publications related to alcohol displays on social media, plus additional investigators with experience in adolescent substance use and social media use, communication neuroscience, and behavioral science.
- The behavioral science expertise is provided by a consultant who is top of the field in this area and will assist with the social media data collection codebook.

Weaknesses

- The assessments of health behavior (diet and physical activity) are weak calling into question the ability of the team to gather, analyze, and interpret these health behavior data that seem outside of the wheelhouse of the other investigators. Expertise in measurement and interpretation of physical activity and diet would be an important addition to the team to address the aims.

3. Innovation:

Strengths

- The focus on the role of both creating and viewing other-generated content on TDM is a particular innovation as this has ecological validity for how adolescents use social media yet is largely lacking from the evidence base.
- The use of the self- and other-generated content from the first aim and applying this to the third aim to show these images to the adolescent during MRI scans is innovative and lends additional ecological validity.
- There is innovation in aim 2 to use EMA to attempt to estimate adolescents' TDM use and social media use through a triangulation of data sources.

Weaknesses

- The adolescent period is often investigated as a time for risky behavior.
- While most research does focus on screen time rather than quality/context, the use of one measure (the ADTI) to measure quality does not seem particularly innovative or sufficient.

4. Approach:

Strengths

- Efficient enrollment of n=400 adolescents across Wisconsin, with shared participant pool across the 3 projects and recruitment ambassadors to assist.
- Appropriate statistical analysis with adequate power to test hypotheses.

- Consideration of potential problems and alternative approaches, including ability to collect the data of interest.
- The study focuses on the 4 currently most popular social media platforms for adolescents but nods to the ability to add more social media platforms if needed during the course of the trial, given the rapid changes in this landscape (e.g., TikTok only recently became popular).
- The statistical approach using latent class analysis is appropriate.
- The protection of risks is well considered and based on prior data collected from the study team regarding adolescents' views of privacy of their online content and willingness to share images/information with the research team. There are appropriate safety guidelines in place in the event self-harm, abuse, or other emergency situations are viewed by the research team.
- The moderators are pertinent and well considered including parental involvement related to household media rules and the parent-adolescent relationship, as well as the self-rated technology importance scale completed by the adolescent.
- EMA will help reduce recall bias. Participant compensation is high for EMA responses which should help with compliance.
- Strong recruitment and retention plan including Recruitment Ambassadors to get connected to youth-serving organizations/schools and a youth advisory group for ongoing input.

Weaknesses

- Overall, how will researchers separate adolescent perception from reality – i.e., both their posting of content and their responses to self-report behavior surveys are subject to social desirability bias – what is their actual behavior vs. what they are projecting towards the online community, their friends, and the research team?
- Several theories are presented to explain TDM influence on adolescent behavior (e.g., theory of reasoned action, social learning theory, media practice model) but it is not clear how these theories informed the research questions, constructs/assessments under study, or hypotheses, or what the overall conceptual model is.
- The recruitment and retention plans are underdeveloped. The sample is projected to be 76% white and all from Wisconsin – how will these results generalize to other race/ethnic groups and regions?
- For aim 1, health behaviors are measured as 1 dietary recall and one 3-item self-report physical activity scale at each timepoint. Adhering to gold standards e.g., 3 dietary recalls including at least 1 weekend day plus accelerometry or similar objective measurement of physical activity would provide more precision for these outcome measures. If actigraphy or similar device is not feasible, even a more robust instrument that can provide a gradient of physical activity beyond dichotomizing the sample would be useful. It is also not clear how the dietary recall will be analyzed e.g., healthy eating index, food groups, calories, macro/micronutrients, etc.
- How will codebooks be altered for video content, e.g., from TikTok and Facebook videos?
- For aim 2, It is not clear why 5-days of EMA was selected (e.g., rather than 7 days) and if adolescents will be able to respond to prompts throughout the day e.g., during school or other activities.
- The EMA response requirements seem challenging to remember the codes/order for response, though the researchers do provide some preliminary data indicating feasibility. Why not use a drop-down menu or closed-button response to the questions? More information on the preliminary EMA feasibility trial is needed – is this a similar population as will be enrolled in the proposed study, were other measures utilized in that study or only EMA? EMA compliance is

often lower in trials that involve multiple burdens on participants vs. those that only employ EMA.

- How will missing data be managed in the EMA aim 2 analyses?
- Aim 3 is innovative yet appears underdeveloped in terms of feasibility of gathering enough persuasive content for each adolescent to show to them in the fMRI scan from their own self-generated posts. It is described that the adolescent assists the researcher in selecting these images, but will this somehow bias the response since the adolescent knows what to expect in the scanner and can select based on preference, impression management, etc. which images to use? How are the other-generated images selected or created since this does not appear to be data/images captured by the research team from the adolescents' own profiles. Does there need to be some standardization across participants, e.g., if some adolescents see all images of alcohol use vs. others seeing other substances?
- How will aim 3 proceed if an adolescent has not posted persuasive content related to health or risk behaviors?
- Appropriate data safety and ethical safeguards are considered for protecting adolescents' privacy. However, additional information on how or whether this will be shown to the parent is important to consider both from an ethical standpoint for adolescent's privacy as well as to avoid social desirability bias or the adolescent hiding things from their parent especially regarding risk behavior/substance use. At the same time, the parent may request to view images or learn about their child's risky behaviors and social media behaviors – how will this be managed?
- What is the plan for adolescents who post no images of substance use or of health behaviors during the study time period, both in terms of the aim 1 and 2 analysis as well as images to show during aim 3? How often is this expected to occur?
- How might time of year, seasonality, school vs. out-of-school impact results?
- What is the rationale for excluding participants who are taking psychoactive medications, and how might this limit generalizability?

5. Environment:
Strengths

- Excellent facilities to carry out the proposed research.
- Strong institutional support including a contribution of $500,000 in matching funds by the investigators' departments.

Weaknesses

- With the cohort-style recruitment, it is not clear if the facilities & staff can handle the bolus of measurements e.g.: fMRI scans needed at each time point.

Protections for Human Subjects

- Acceptable Risks and/or Adequate Protections
- Considerations made for confidentiality and participant privacy, as well as to mitigate safety issues.

Data and Safety Monitoring Plan (Applicable for Clinical Trials Only):

- Acceptable
- Data safety considered particularly related to participant confidentiality/privacy.

Inclusion of Women, Minorities, and Individuals Across the Lifespan

- Sex/Gender: Distribution justified scientifically
- Race/Ethnicity: Distribution justified scientifically
- For NIH-Defined Phase III trials, Plans for valid design and analysis: Scientifically acceptable
- Inclusion/Exclusion of Children under 18: Including ages <18; justified scientifically
- Expected 76% White sample to mirror the U.S. census; does not appear to be attempts to increase diversity of sample.

Vertebrate Animals

- Not Applicable (No Vertebrate Animals)

Biohazards

- Not Applicable (No Biohazards)

Resource Sharing Plans

Acceptable

- Data shared through the NICHD DASH.

Budget and Period of Support

Recommend as Requested

PROJECT 1, CRITIQUE 3:

Significance: 3
Investigator(s): 2
Innovation: 5
Approach: 3
Environment: 1

Overall Impact:

This first project of this P01 plan entails a series of descriptive experiments that hold potential to relate the social media activity and content perusal of adolescents, especially content related to substance use and other health behaviors, to their actual self-reported health behaviors and attitudes. Moreover, the study holds potential to examine the relative rates at which different adolescents engage their own generated content versus the content of others, and how those exposures relate to health behavior. Finally aim 3 will use functional magnetic resonance imaging to determine whether recruitment of valuation areas of the brain by self-related content as captured from the participants device as well as other, sourced content relates to self-reported health behavior. The team is extremely strong and has demonstrated amply the likely feasibility of the data collection proposed based on previous work, including orderly schemes for coding and rating participant generated content. The team proposes to use validated self-report metrics of health behaviors. Enthusiasm was somewhat dimmed however, by the very vague statistical plans and lack of clarity in the specific plans to capture and present other-

sourced content. In addition, the functional MRI analytic plan and conceptualization makes no reference to the abundant literature on recruitment of self-referential neural circuitry itself, and how that may dovetail or otherwise interact with valuation circuitry. Finally, preliminary data offered minimal proof-of-concept, and were suggestive only of elements of feasibility. For example, we have no information on how often teens will truly report themselves exercising, if enough for analysis. However, the strengths of the application outweigh its weaknesses.

1. Significance:
Strengths

- How does social media usage and user-generated content relate to health behavior in younger adolescents is a significant knowledge gap this study could fill
- Taking a page from the field of “neuromarketing” of other-generated content (ads) and applying it to peer/other-related content to assess recruitment of motivational neurocircuitry holds potential to uncover brain mechanisms for TDM-mediated peer influence

Weaknesses

- Lack of clarity in how other-sourced content is composed (text? Images?) and presented in Aim 3 make ascertainment of its significance a tad compromised.
- Lack of clarity in fundamental metrics of TDM immersion in different categories of content also diminish significance

2. Investigator(s):
Strengths

- The team has expertise in different elements of TDM capture
- Team has expertise in relationship between social media use and health behaviors

Weaknesses

- The team has no expertise in the functional imaging of self-referential vs other-referential content

3. Innovation:
Strengths

- Adolescents have been scarcely studied on TDM use in this level of detail
- Use of multiple TDM/social media platform
- Use of novel text-based EMA capture of participant’s self-reported TDM immersion and content
- Use of fMRI to probe the motivational “traction” of health-related content derived from TDM is novel
- Inclusion of positive health behaviors in analysis

Weaknesses

- Friending participants to monitor their use and posts is pretty industry-standard

4. Approach:
Strengths

- Use of a reasonably validated EMA approach to capture what content a participant is partaking in, in terms of other-generated content
- Solid fMRI preprocessing and metrics
- Solid evidence of successful TMD usage and content capture in team's previous work

Weaknesses

- Minimal pilot data on proof of concept; pilot data pertained primarily to feasibility/retention
- In all analyses that purport to quantify TMD self-generated content engagement, the details are vague, such as whether engagement is defined as proportion of EMA pings in which that content was engaged vs total times that type of content was engaged, or whether for example, frequency of posting unhealthy behaviors is indexed by total posts per unit time or as a fraction of total content presented on their personal platform
- In Aim 2, not clear what and how participant is to code text response if they are not looking at anything related to ABCD or E
- Aim 2 unclear why self-reported EMA responses are explored as index of total TDM usage when there are plenty of apps out there that track screen time (with no recall bias), e.g., EARS (https://ksanahealth.com/ears/)
- Potential pitfalls paras basically assert that there will likely be no pitfalls, and so no actual alternative approaches (e.g., increasing stipends or swag if dropouts are greater than expected) are ever offered
- It is unclear how other-generated content is captured to be presented in the scanner for Aim 3 (supposedly from Aim 2), since there is no plan e.g., for participants to donate favorite screen captures they could grab from other-sourced content. (Unless this content is actual text-based)
- No consideration of brain-wide search for differences between self- vs other-generated content
- Not clear why fMRI analytic plan fractionates trials into different categories (compromising n of trials/events) based on persuasiveness, when they could just amplitude-modulate each stimulus based on the 1-4 scale of its persuasiveness in the regression model

5. Environment:

Strengths

- UW-Madison has the needed infrastructure, especially considering the low computational needs
- Already demonstrated web-based text platform capacity

Weaknesses

- None Noted

Protections for Human Subjects

- Acceptable Risks and/or Adequate Protections
- The team has a reasonable plan/threshold for breaking confidentiality in instances of suicidal ideation or other grave health risks

Data and Safety Monitoring Plan (Applicable for Clinical Trials Only):

- Not Applicable (No Clinical Trials)

Inclusion of Women, Minorities, and Individuals Across the Lifespan

- Sex/Gender: Distribution justified scientifically
- Race/Ethnicity: Distribution justified scientifically
- For NIH-Defined Phase III trials, Plans for valid design and analysis: Not applicable
- Inclusion/Exclusion of Children under 18: Including ages <18; justified scientifically
- This is an adolescent-focused study

Vertebrate Animals

- Not Applicable (No Vertebrate Animals)

Biohazards

- Not Applicable (No Biohazards)

Resource Sharing Plans

- Acceptable

Budget and Period of Support

- Recommend as Requested

PROJECT 2: USING FUNCTIONAL MAGNETIC RESONANCE IMAGING TO UNDERSTAND HOW POSITIVE AND NEGATIVE TDM EXPERIENCES RELATE TO MENTAL AND BEHAVIORAL HEALTH.
Principal Investigator: Dr. Christopher Cascio
Overall Impact Score: 55

(Description as provided by applicant): Social connectedness and inclusion are essential for well-being and health, particularly during adolescence, a period characterized by an increased salience placed on peer relationships and experiences. Technology and digital media (TDM), particularly social media, has the affordance of connecting with peers. In fact, TDM has become an increasingly important form of communication for adolescents, 71% of adolescents report using more than one social media site and 92% of adolescents between the ages of 13-17 years old report going online daily. Reviews on TDM use, however, suggest that there is not consensus on the impact of TDM and social media use on well-being and health behaviors among adolescents, finding positive, negative, and null results. They highlight that little evidence exists that examines potential moderating factors between TDM use and well- being and health outcomes, which limits our understanding of what influences outcomes of interest. Thus, there is an urgent need to fill this gap. Effects associated with TDM use may depend on specific ways that adolescents use TDM. For example, if high levels of exposure to certain kinds of TDM, such as social media, sensitize adolescents to other people's opinions, they may be more reactive to negative experiences such as social exclusion, but may benefit more from experiences of social inclusion. This sensitivity may help explain the mixed findings within the literature. The current study proposes to test this idea by measuring adolescent's exposure to social media (drawing data from Projects 1 and 3) that have the affordances of social connectedness (i.e., likes, comments, number of friends or followers). Then we will relate these experiences longitudinally to their reactivity to experiences of social inclusion and exclusion to predict health and risk behaviors as well as well-being

(drawing data from Projects 1 and 3) and examine how changes in functional reactivity across the social transition period from middle to high school, a period associated with changes in social ties, risk-taking, and challenges to well-being, may influence these relationships. This data will not only examine deficits associated with social media use that led to poorer well-being and unhealthy behaviors but will also examine the strengths of social media use and how they relate to better well-being and healthy behaviors. Further, if adolescents are sensitized to social cues, the data can help identify ways to help steer adolescents towards social media engagement that leads to greater well-being and healthier behaviors. This project aligns with the current RFA as it tests how TDM usage affects health behavior. This project specifically aligns with two areas of interest for this RFA: 1) interdisciplinary studies of TDM usage across adolescence employing multi-level assessments of neurodevelopment to examine interrelated developmental changes in brain function and complex behavior, and 2) real-time measures of TDM exposure and usage and how this usage regulates adolescents' behavior.

PROJECT 2, CRITIQUE 1:

Significance: 3
Investigator(s): 2
Innovation: 3
Approach: 4
Environment: 2

Overall Impact:

This project will utilize a well characterized game for functional magnetic resonance imaging called cyberball to examine wither reactivity of the brain's salience network (composed of anterior insula and dorsal anterior cingulate cortex) to social exclusion or inclusion relates cross-sectionally to real world risk behaviors, and whether this brain reactivity in turn moderates the relationship between social media engagement and positive or negative health outcomes. In addition, repeated testing with the cyberball task for functional MRI will be used to determine whether changes in brain reactivity to exclusion or inclusion over time (transition from middle school to high school) relates to positive or health negative health outcomes, as further moderated by whether the social media experience in the intervening interval is positive or negative. A key strength and point of significance of this project is that it will ostensibly examine individual differences moderators of the relationship between social media engagement and emotional and risk behavior outcomes. Thus, it holds potential to resolve why some studies, or individual adolescent participants fare better with more social media engagement, while others fair worse. Other strengths include a well characterized task of social exclusion, and an investigative team that has collective experience in collection of all data modalities, not only the functional MRI signal, but also metrics of social media engagement and features of the social media experience. However, enthusiasm was dimmed by the absence of any data either from other literature or preliminary data from the team on the longitudinal stability and reliability of salience network recruitment by cyber ball inclusion or exclusion. The field of task fMRI in general is undergoing a critical evaluation considering recent evidence that even well understood task activations in commonly replicated regions show very lackluster test-retest reliability. This calls into question the ability for aim 3 in particular to be borne out. Another concern is the lack or assumption in aim 3 that changes within subject in reactivity over time are moderated by the positive versus negative experience of social media as an ostensibly orthogonal phenomenon, when the changes in brain reactivity by exclusion themselves may be driven by intervening social media valence. In addition, the hypothesis in specific Aim 2 presumes that greater reward reactivity during inclusion is a marker of emotional resilience when this assumption may not be true in that greater reward reactivity may simply reflect an unhealthy

dependence on social reinforcement or may represent an escape (relief) from negative evaluations. In general, there were more strengths than weaknesses in this proposal.

1. Significance:

Strengths

- Understanding why some kids do better emotionally with increased social media engagement and others do worse, in terms of potentially moderating individual-difference factors, is poorly understood.
- Social media engagement can be a significant risk factor for some kids, where understanding what moderating factors exist as targets (such as hypersensitivity to affirmation) would enable targeting of those attitudes/assumptions in psychotherapy is plausible

Weaknesses

- Aim 1 cross-sectional relationship between salience network (SN) reactivity to exclusion and negative health behaviors has ostensibly already been reported in cited literature. The applicants do not make a case for what this aim would add
- The premise that greater striatal responsiveness (e.g., reward activation) by social inclusion is a resilience marker is challenged not only by the lack of preliminary data on this valence, but also by the possibility that such responsiveness may indicate an unhealthy dependence on positive reinforcement, or simply a relief from exclusion, such as if the inclusion block occurred last in the sequence, following the exclusion block.

2. Investigator(s):

Strengths

- The team has collective expertise in social fMRI (e.g., Dr. Tezler, Dr. Cascio)
- The team has experience in quantification of social media (valence) and metrics

Weaknesses

- None Noted

3. Innovation:

Strengths

- Understanding temperamental individual-difference moderators of adolescent reactivity to social media immersion is novel
- Understanding neurocircuit-based individual-difference moderators of adolescent reactivity to social media immersion is especially novel

Weaknesses

- Aim 1 cross-sectional relationships between brain reactivity to social inclusion and exclusion and real-world risk-taking has already been demonstrated to some extent

4. Approach:

Strengths

- Use of fMRI to probe covert valuation of social stimuli may get around poor self-report due to investigator-pleasing or simple lack of insight/metacognition in some teens.

- Cyberball exclusion-elicited activation of salience network (also “pain network” as one type of salient stimuli) has shown relationships to real-world risk-taking for social affirmation, making many proposed findings plausible.
- Use of confederates in the second application of cyberball will enhance plausibility
- Careful tracking of multiple social media content without intrusiveness
- The longitudinal interval of 18 months, while spanning the change from middle school to high school maximizes social milieu change while minimizing the test-retest interval and related dropout.

Weaknesses

- Test-retest reliability of cyberball has not been established, and the power analysis section does not clearly indicate if this has been ascertained. In general, the field is now coming to grips with how reliabilities within-subject of contrast-elicited task-fMRI metrics, even in well-characterized Region of Interest (ROI) are poor, capping out at .5 even at much shorter inter-scan intervals (c.f. PMID 32489141). This calls into serious question how valid the change in ROI signal value by exclusion/inclusion from T1 to T2 within-participant will be.
- Not clear how inclusion-related activation/signal is distinctly indexed, when ROI signal is generally the contrast between exclusion block signal minus inclusion block signal (pain in SN) or inclusion minus exclusion (reward in VS).
- Assuming test-retest reliability of cyberball is unusually robust, aim 3 approach assumes that change in limbic reactivity is a marker in itself to relate to risk behaviors that is ostensibly independent from the general positivity of social media signals in the interim between scans (as a proposed moderator) when in fact this positivity may be what drives brain reactivity change. In general, inclusion of this valence moderator was kind of a distraction, when even the core relationship between within-subject change and risk/health outcomes themselves would have been interesting enough.
- Contrary to applicant claims of non-interdependence of Aims, aims 2 and 3 are still somewhat contingent on Aim 1 replicating previous findings that the SN response to exclusion still has something to do with real-world risky behavior or with self-reported need for social affirmation or belongingness. However, this is only a minor concern.

5. Environment:

Strengths

- UW-Madison has all the scanning capacity needed
- Social media monitoring and questionnaire collection do not require elaborate infrastructure

Weaknesses

- None Noted

Protections for Human Subjects

- Acceptable Risks and/or Adequate Protections
- fMRI with no contrast media is low risk
- Social media monitoring will be unobtrusive (no comments on media)

Data and Safety Monitoring Plan (Applicable for Clinical Trials Only):

- Not Applicable (No Clinical Trials)

Inclusion of Women, Minorities, and Individuals Across the Lifespan
- Sex/Gender: Distribution justified scientifically
- Race/Ethnicity: Distribution justified scientifically
- For NIH-Defined Phase III trials, Plans for valid design and analysis:
- Inclusion/Exclusion of Children under 18: Including ages <18; justified scientifically
- Suitable population age-wise

Vertebrate Animals
- Not Applicable (No Vertebrate Animals)

Biohazards
- Not Applicable (No Biohazards)

Resource Sharing Plans
- Acceptable

Budget and Period of Support
Recommend as Requested

PROJECT 2, CRITIQUE 2:

Significance: 5
Investigator(s): 6
Innovation: 3
Approach: 6
Environment: 2

Overall Impact:

This research project examines important questions about how reactivity to social exclusion and inclusion relates to the transition to high school and to social media experiences, and how reactivity to social exclusion and inclusion might mediate the relation between social media engagement and health outcomes. Novel aspects of the proposed work include using fMRI as a physiological measure of reactivity to social exclusion and inclusion and examining changes in reactivity over time in relation to the transition to high school and social media engagement. The application falls far short of its goals because the conceptualization is weak and not theoretically informed, it is not clear how exactly how participants for the fMRI project will be recruited and how representative the sample will be, more information is needed about how social media engagement will be captured and coded, and the team could benefit from the addition of a statistical consultant to develop an analytic plan to address the specific aims.

1. Significance:
Strengths
- Examining sensitivity to social exclusion and inclusion using fMRI could elucidate why social media experiences affect individual youth in distinct ways.
- Investigating how sensitivity to social exclusion and inclusion change in relation to school transitions and social media experiences could inform our understanding of how social experiences affect reward and pain centers of the brain.

Weaknesses
- The project is poorly conceptualized. Filling gaps is not interesting and important. It is not clear how these results will inform theoretical models or prevention/intervention approaches.
- This project does not carry through with the strong emphases in the parent application on social media as an opportunity for identity development and peer connection.
- This application does not cite a wealth of relevant research and theory that could provide a much stronger conceptual framework for this investigation.
- Specific Aim 1 does not relate to Technology and Digital Media Use.
- The direction of causal relationships and possible mechanisms are not clearly elucidated.

2. Investigator(s):
Strengths
- The Project Lead, Dr. Cascio, has strong expertise in how neural sensitivity relates to social influence.
- The Co-Investigator, Dr. Telzer, has strong expertise in longitudinal, neuroimaging studies of adolescence, an impressive scholarly record, and experience in studying neural correlates of adolescent peer relationships.

Weaknesses
- The PI is junior, only three years post-Ph.D., with no experience leading this type of project and little expertise on adolescents' engagement with social media.
- The PI and the Co-I have not previously collaborated; it is not clear that they can work together to carry out this complex project.
- This team does not have the statistical expertise to carry out this research.

3. Innovation:
Strengths
- Examining reactivity to social exclusion and inclusion using fRMI is innovative.
- Examining how physiological reactivity to social exclusion and inclusion relate to the transition to high school and to social media experiences is novel.
- Examining how reactivity to social exclusion and inclusion might moderate the relation between TDM and health outcomes could inform theories of why some youth experience more harm and benefits than others.

Weaknesses
- Filling gaps is not innovative.

- This study is not theoretically motivated; thus, it is not clear how it could enhance theoretical models.
- The application barely cites previous research, thus does not make clear precisely how this study extends earlier work.

4. Approach:
Strengths

- The longitudinal design allows examination of changes in reactivity to social exclusion and inclusion over time, and in relation to the transition to high school and to social media exposure.
- Using fMRI to measure reactivity to social exclusion and inclusion while participants play Cyberball has the potential for ecological validity.

Weaknesses

- Although we are reassured throughout this application that Recruitment Ambassadors will assist with recruiting participants, it is not clear exactly how this will be done for the fMRI study and how representative the resulting sample will be.
- The application does not cite any previous research or include pilot data showing that Cyberball is feasible to measure reactivity to social inclusion and social exclusion using fMRI.
- Gathering data on social media by friending participants and accessing their profiles does not allow access to private messaging that has found in previous research to be more strongly related to substance use than public posts, and it does not allow archiving and saving the data. The application provides insufficient detail on how precisely social media will be collected and over what amount of time.
- Much more detail is needed about how the content of social media will be coded, and how the dizzying number of variables to be examined across the four different platforms will be treated in the analyses.
- Although some information is provided about how the fMRI data will be analyzed, there is no analytic plan describing the statistical techniques for examining Specific Aims 1 – 3. In the application where the analytic plan typically appears, the aims are simply repeated.

5. Environment:
Strengths

- The University of Wisconsin and the University of Chapel Hill offer rich institutional support, equipment, and other physical resources to support this project.
- The sharing of one large participant pool with other projects from this Center application make this research more feasible.

Weaknesses

- The application could be more specific about how this project would benefit from unique features of these scientific environments.

Protections for Human Subjects

- Unacceptable Risks and/or Inadequate Protections

- It is not clear how the confidentiality of social media data will be protected. Social media data cannot be easily de-identified, so more information is needed about how it will be archived, coded, and stored.

Data and Safety Monitoring Plan (Applicable for Clinical Trials Only):

- Not Applicable (No Clinical Trials)

Inclusion of Women, Minorities, and Individuals Across the Lifespan

- Sex/Gender: Distribution justified scientifically
- Race/Ethnicity: Distribution justified scientifically
- For NIH-Defined Phase III trials, Plans for valid design and analysis: Not applicable
- Inclusion/Exclusion of Children under 18: Including ages <18; justified scientifically
- The focus on adolescents in scientifically appropriate.

Vertebrate Animals

- Not Applicable (No Vertebrate Animals)

Biohazards

- Not Applicable (No Biohazards)

Budget and Period of Support

- Recommend as Requested

PROJECT 2, CRITIQUE 3:

Significance: 6
Investigator(s): 5
Innovation: 8
Approach: 7
Environment: 2

Overall Impact:

Project 2 focuses on a subset of n=150 participants who will come to the imaging center for fMRI scans twice over the course of the grant, at ages 13/14 and two years later at 15/16 years. Specifically, neural activity in reward processing brain regions during social inclusion (change from exclusion block), and neural activity in social pain brain regions during social exclusion (change from inclusion block) will be measured using the Cyberball paradigm. During Cyberball, the participant plays catch with 3 other cyber players (i.e., the computer) – where they are included, then excluded. Aim 1 involves using these fMRI measures at Time 1 to predict concurrent and longitudinal health risk outcomes. Under Aim 2, the fMRI measures are used as moderators of the association between social media use (from Project 3) and health risk outcomes. Finally, with Aim 3, the relation between longitudinal changes in the fMRI measures and longitudinal changes in positive/negative peer interactions on social media use will be examined. The importance of technology, parent involvement, and health risk behaviors will also be considered as moderators. There are two subcontracts to UNC-Chapel Hill and UPenn for consultation

with more experienced investigators. The study focuses on an important developmental period for social inclusion/exclusion and the investigators have relevant experience and are in a supportive environment; however, there are also several significant limitations.

Weaknesses of the study include an unclear review of the relevant extant literature. Preliminary studies of social exclusion and risky behavior and Facebook friendship networks are described well, but a summary of the number of studies that find these associations and relevant effect sizes are not specified. Further, the study by co-I Tezler showed that the target reward processing centers of the brain were associated with peer conflict, rather than well-being, which seemed to contradict the current hypotheses. In terms of innovation, there were no new theory, methods, instrumentation, or interventions proposed. The PI has the relevant training but is a new PI with no experience managing a research study with external funding. There are also several issues with the approach. Social exclusion and social inclusion are operationalized as difference scores based on the other, e.g., social exclusion > social inclusion in social pain brain regions. With this difference score approach without the use of a true baseline, it is unclear whether social exclusion or social inclusion or both are contributing to the measures. Second, an effect size of 0.30 was used in the power analysis with no evidence that this is appropriate for reactivity in these brain regions based on previous literature. Third, no evidence was given that 150 of the 400 participants would pass the fMRI exclusion criteria, e.g., no history of psychiatric disorders, yet greater than 50% of adolescents in general have clinical levels of anxiety/depression (with rates even higher among LGBTQ+ youth). In addition, comments from the participants' social media friends will be viewed and coded. What is the protocol if friends reveal harm to self or other? Lastly, the outcome measures tap narrow constructs that do not fully capture "health, risky behaviors, and well-being." For example, health is only measured with self-report of frequency of exercise (3-items) and a 24-hour food recall. Risky behaviors are only measured with attitudes, intention, and use of drugs and alcohol. Well-being is operationalized as perceived stress, loneliness, peer problems, and low self-esteem—yet a lot was made of assessing positive outcomes. Because of these limitations, the overall impact is likely to be modest.

1. Significance:
Strengths

- PI Dr. Cascio and co-investigator Dr. Falk have shown that increased neural reactivity in social pain regions during social exclusion has predicted increased risk-taking in the presence of peers.
- PI Drs. Cascio and Falk found social exclusion brain region reactivity related to Facebook friend density and transitivity in male adolescents.
- Co-I Dr. Tezler found that found that reactivity in affective and reward processing brain regions was associated with peer conflict, especially with low peer support.

Weaknesses

- Unclear scope of the previous literature, Significance and Preliminary Studies focus on previous study of social exclusion and risky behavior or Facebook friendship networks, but effect sizes and number of studies that find these associations are not specified.
- Reward processing centers of the brain were associated with peer conflict (Dr. Tezler studies), which is not in line with current hypotheses.

2. Investigator(s):
Strengths

- PI Dr. Christopher Cascio, Assistant. Professor at UW, Journalism and Mass Communication.

- Co-I Dr. Dhavan Shah will help design the study and is expert on the influence of electronic/digital media on social judgments.

Weaknesses

- Subcontract co-investigators could be budgeted as consultants as they have limited support (about two weeks) and their purpose is to give expert advice.
- PI Dr. Cascio has no experience with external funding. Arrived at UW Madison in July 2021.

3. Innovation:

Strengths

- fMRI approach to investigate reactivity to social exclusion and inclusion is innovative.

Weaknesses

- Innovation section continued to explain the specific aims, with no new theory, methods, instrumentation, or interventions.

4. Approach:

Strengths

- Extensive assessment: Social media use: Every month, a 1-week sampling period of all content on the participant's social media feed will be downloaded for analysis, for a total of 24 one-week sampling periods across two years. Number of likes, comments, friends/followers, and shares will be coded.
- Network interaction data is also downloaded expression and reception.
- Participants will complete a measure of social inclusion and exclusion using Cyberball in the MRI. The Cyberball task will consist of examining neural activity in the contrast in social pain brain regions (social exclusion > social inclusion); and social inclusion compared to social exclusion in reward brain regions (social inclusion > social exclusion).
- All fMRI participants will be right-handed, not suffer from claustrophobia, no psychoactive medications, no history of psychiatric/neurological disorders, normal (or corrected to normal) vision, no metal, and not currently be pregnant.

Weaknesses

- Social exclusion and social inclusion are operationalized as difference scores based on the other, e.g., social exclusion > social inclusion in social pain brain regions. With this difference score approach without the use of a true baseline, it is unclear whether social exclusion or social inclusion or both are contributing to the measures.
- Power: an effect size of 0.30 was used with no evidence that this is appropriate based on previous literature on activation in these brain regions.
- What is the evidence that 150 participants will meet the fMRI exclusion criteria, e.g., no history of psychiatric disorders, yet greater than 50% of adolescents have clinical levels of anxiety/depression (raters even higher among LGBTQ+ youth)?
- Social media coding is limited to 4 platforms: Facebook, Twitter, Instagram, and TikTok, yet Facebook and Twitter are not frequently used by adolescents to interact with their peers. How will analyses be adjusted for platform? Direct messages are excluded.

- Only one task is used to measure social inclusion and exclusion – Cyberball. The approach would be strengthened by measuring these constructs using multiple paradigms, as longitudinal validity of Cyberball is poor.
- Outcome measures tap narrow constructs using only self-report. Health, for example, is only measured with self-report of activity (3-items—frequency of exercise), and 24-hour diet/nutrition (excellent measure). There are many other aspects of health that could be affected. The positive side of well-being is not adequately assessed.

5. Environment:
Strengths

- The University of Wisconsin Waisman Center MRI scanner is a 3 Tesla (3T) GE MR750 scanner. There is a lot of support for fMRI work at the Waisman imaging center.

Weaknesses

- Unclear why subcontracts are needed to UPenn and UNC-Chapel Hill when co-I's are solely advising/consulting at approximately 2 weeks/year.

Protections for Human Subjects

- Unacceptable Risks and/or Inadequate Protections
- Most safeguards are in place, but it was unclear what would be done if participants' social media friends disclosed harm to self or other. More information on how youth's confidentiality around social media content is needed.

Data and Safety Monitoring Plan (Applicable for Clinical Trials Only):

- Not Applicable (No Clinical Trials)

Inclusion of Women, Minorities, and Individuals Across the Lifespan

- Sex/Gender: Distribution justified scientifically
- Race/Ethnicity: Distribution justified scientifically
- For NIH-Defined Phase III trials, Plans for valid design and analysis: Not applicable
- Inclusion/Exclusion of Children under 18: Including ages <18; justified scientifically
- 50% female, 18.5% Hispanic/Latino, 76% White, 13% Black, 6% Asian, 2% American Indian/native Hawaiian or Pacific Islander, and 3% more than one race

Vertebrate Animals

- Not Applicable (No Vertebrate Animals)

Biohazards

- Not Applicable (No Biohazards)

Resource Sharing Plans

Acceptable

- All 3 Project PIs will plan to share data through NICHD's DASH

Budget and Period of Support

- Budget Modifications Recommended (in amount/time)

Recommended budget modifications or possible overlap identified:

- Subcontract co-I's could be budgeted as consultants as they are solely advising.

PROJECT 3: USING MIXED METHODS TO EVALUATE SELF- AND OTHER-GENERATED TDM CONTENT AS PREDICTORS OF SOCIOEMOTIONAL WELL-BEING IN SEXUAL AND GENDER MINORITY (SGM) AND NON-SGM ADOLESCENTS.
Principal Investigator: Dr. Ellen Marie Selkie
Overall Impact Score: 36

(Description as provided by applicant): The development of socioemotional well-being in adolescence is crucial for mental and physical health. Technology and digital media (TDM), and especially social media, are highly used among adolescents and hold risks and opportunities for socioemotional well-being. These risks and opportunities may be more pronounced in sexual and gender minority (SGM) adolescents. The long-term goal of our research is to promote well-being for adolescents and young adults by decreasing negative social media activities and amplifying positive social media activities. The objective of this Project is to understand the types of social media content that adolescents create (self-generated) and consume (other-generated), and how these social media activities affect socioemotional well-being. The Aims are: 1) To test bidirectional relationships between self-generated TDM content and socioemotional well-being; 2) To test bidirectional relationships between other-generated TDM content and socioemotional well-being; and 3) To compare the self-and other-generated TDM experiences of sexual and gender minority (SGM) adolescents with those of non-SGM adolescents. The PI, Co-Is, and Consultant on this Project bring decades of research experience with adolescents and social media. The PI's preliminary work has included development of a novel ethnographic approach to directly observed self-generated social media content and accumulated experience with longitudinal study design in early adolescents. The proposed Project will use longitudinal data collection over 2 years. The shared participant pool for this P01 program will be leveraged to recruit the sample of 400 adolescents aged 13-15 years at enrollment. Data collection approaches included online ethnographic observation of participants' self-generated social media content, thematic analysis of other-generated content experienced by participants, individual interviews, and survey administration. These data will be used to test a cross-lagged panel model examining socioemotional well-being as a predictor and outcome of self-generated and other-generated social media experiences over time. Qualitative comparison groups will be used to compare self-and other-generated social media experiences between SGM and non-SGM adolescents. This Project aligns with the current RFA as it tests how TDM usage impacts social-emotional development and health outcomes in adolescents from diverse backgrounds and subpopulations.

PROJECT 3, CRITIQUE 1:

Significance: 1
Investigator(s): 1
Innovation: 2
Approach: 2
Environment: 1

Overall Impact:

This research project is a study of socioemotional well-being and TDM use among SGM and non-SGM youth. The project uses mixed methods, including ethnography and survey research. The investigator is also lead of the R&R core, which should facilitate recruitment and data collection. The investigative team is strong overall, the project is significant novel in many respects, including the “total marketplace” focus on creation and consumption of social media, especially by SGM youth. Approach is strong, with minor weaknesses related to retention and power analysis. Overall strengths greatly outweigh weaknesses in this potentially impactful research project that integrates well with the overall program project and other cores and research projects.

1. Significance:
Strengths

- Focus on the “total marketplace” of both creation and consumption of social media content is significant
- Project aligns well with the overall Brain, Behavior, and well-Being conceptual framework of the P01
- Measures and analysis of both active and passive social media use/consumption is a strength
- Focus on social and emotional well-being is significant, especially among SGM youth
- Focus on in-depth analysis of social media content creation is significant

Weaknesses

- None noted

2. Investigator(s):
Strengths

- Dr. Selkie is well positioned to serve as PI
- The inclusion of Dr. Schulenberg as consultant is a strength
- Inclusion of Dr. Ranney and Brown collaboration is a strength
- Dr. Toma provides statistical analysis expertise

Weaknesses

- None noted

3. Innovation:
Strengths

- Focus on ethnographic methods, younger adolescents (age 13-15), and upstream outcomes are novel
- Focus on bidirectional relationships between social media content and socioemotional well-being is novel

Weaknesses

- Longitudinal methods are not especially novel
- It would’ve been helpful for this section to be tied more directly into the conceptual framework for the overall P01, more discussion of how the project innovates within the framework (noting that there is some discussion of conceptual framework specific to this project in the approach, but how is it integrated)

4. Approach:
Strengths
- Integration of project recruitment plan with R&R core (Dr. Selkie also PI of that core) is a strength
- Social media platforms and data collection plan is well-articulated and a strength
- Clear timeline and ongoing social media data collection plan
- Ethnographic methods are a strength
- Focus on social media content creation and in-depth analysis are strengths
- Mixed methods (interviews, questionnaires) are a strength and well justified
- Data analysis plan is a strength

Weaknesses
- Power analysis is only briefly mentioned, more detail and justification for sample size needed (minor)
- Possible issues with retention are not fully addressed, more detail needed

5. Environment:
Strengths
- UW environment, and Dr. Selkie's team and research setting, are excellent
- Description of the Brown University collaborating environment is very good

Weaknesses
- None noted

Protections for Human Subjects
- Acceptable Risks and/or Adequate Protections
- Acceptable

Data and Safety Monitoring Plan (Applicable for Clinical Trials Only):
- Not Applicable (No Clinical Trials)

Inclusion of Women, Minorities, and Individuals Across the Lifespan
- Sex/Gender: Distribution justified scientifically
- Race/Ethnicity: Distribution justified scientifically
- For NIH-Defined Phase III trials, Plans for valid design and analysis: Not applicable
- Inclusion/Exclusion of Children under 18: Including ages <18; justified scientifically
- Justified and acceptable

Vertebrate Animals
- Not Applicable (No Vertebrate Animals)

Biohazards

- Not Applicable (No Biohazards)

Budget and Period of Support

- Recommend as Requested

PROJECT 3, CRITIQUE 2:

Significance: 4
Investigator(s): 2
Innovation: 3
Approach: 4
Environment: 2

Overall Impact:

The aims of this proposal are as follows: 1: To test bidirectional relationships between self-generated technology and digital media (TDM) content and socioemotional wellbeing; 2: To test bidirectional relationships between other-generated TDM content and socioemotional
well-being; and 3: To compare the self-and other-generated TDM experiences of sexual and gender minority (SGM) adolescents with those of non-SGM adolescents.
Strengths include a focus on data collected on self-generated, other-generated and well-being and an exploratory focus on sexual and gender minority teens; a strong and collaborative investigator team with a PI well-poised to do this work and very strong preliminary data. Weaknesses include some lack of clarity on the hypotheses this application puts forth, lack of clarity on any eligibility criteria for participants and the terminology of “offline support systems.” Overall, the strengths outweigh the weaknesses, and this application holds the promise of conducting impactful work.

1. Significance:
Strengths

- The focus on how TDM (social media, in this case) is being used, as opposed to how much it is being used is important and a strength of this proposal.
- This proposal’s focus on linking social media content to the well-being of the individual who created it as well as examining certain types of self-generated content that lead to an improved sense of well-being is a significant aspect of this proposal.
- Collecting data on self-generated, other-generated, and well-being, and an exploratory focus on sexual and gender minority (SGM) teens is a strength.
- The plan to directly examine the benefits to SGM vs. non-SGM youth lends to the significance of this proposal.

Weaknesses

- The hypotheses described are confusing and it is unclear re: how, for example, higher TDM engagement with peers will be predictive of higher socioemotional well-being? Perhaps it is just unclear as to what is meant by “higher?” Higher quality? Higher level of positivity? This lack of clarity on this aspect of the proposal is a weakness.

- Similarly, the following hypothesis, that states: “that consumption of TDM content connected to offline life (higher vs. lower) will be predictive of socioemotional well-being (higher and lower, respectively” is unclear in its reference to “offline life,” as it is confusing as to exactly what is meant by that and then it is under-developed in the rest of the proposal.

2. Investigator(s):
Strengths

- This is a strong investigator team with the requisite experience and expertise to conduct and complete this project.
- Dr. Selkie, the lead, is well-poised to conduct this work as she is in year 4 of an NICHD-funded K23 with great relevance to the proposed work. She has refined the online ethnography and coding procedures, which will be a great asset to the proposed study.
- The other investigators and consultants provide the necessary and complementary skills and experience for this project.
- There is evidence of past and current collaborations within the team which is a strength.

Weaknesses

- No significant weaknesses noted.

3. Innovation:
Strengths

- There are several areas of innovation noted in this proposal, particularly the use of direct observation of adolescent social media activity and data collection at multiple timepoints; this helps to minimize social desirability bias and lack of context.
- The longitudinal design, over the course of 2 years, the bidirectional nature of the examining the data, and the focus on real-life outcomes in terms of teen well-being are novel and important aspects of this study.

Weaknesses

- No major weaknesses noted.

4. Approach:
Strengths

- The combination of the survey component, which includes the focus on socioemotional well-being and the open-ended qualitative interviews, is a major strength to the approach.
- The awareness and sensitivities around the bandwidth of teens to answer survey questions is an important strength in this study design.
- The preliminary studies that serve as the foundation for this proposal are very strong and very relevant to the work being proposed.
- The use of Recruitment Ambassadors is a strength, especially in terms of recruiting underrepresented adolescents.
- A solid rationale is provided for which social media platforms will be used.

Weaknesses

- It is not clear if there are any eligibility requirements re: frequency of social media use given this would be a real limitation to the data collection and findings of this study if there are teens with very limited use.
- There is mention in the Approach re: “Our overarching hypothesis is that adolescents who actively use social media and interact with authentic content related to offline support systems will demonstrate improved socioemotional well-being.” But it is not clear or developed at all as to what “offline” means in this context or what these “offline support systems” are.
- It is not clear in the initial recruitment call (as described in the Approach), if this is simply verbal parental consent and adolescent assent, or when written consent/assent is obtained.

5. Environment:
Strengths

- The environments of the University of Wisconsin as well as the University of Michigan and Brown University, under the umbrella of the environments of the overall project and the other projects, provide excellent support and infrastructure for the conduct of this research.

Weaknesses

- No significant weaknesses noted.

Protections for Human Subjects

- Acceptable Risks and/or Adequate Protections

Data and Safety Monitoring Plan (Applicable for Clinical Trials Only):

- Not Applicable (No Clinical Trials)

Inclusion of Women, Minorities, and Individuals Across the Lifespan

- Sex/Gender: Distribution justified scientifically
- Race/Ethnicity: Distribution justified scientifically
- For NIH-Defined Phase III trials, Plans for valid design and analysis: Not applicable
- Inclusion/Exclusion of Children under 18: Including ages <18; justified scientifically
- Distributions justified scientifically

Vertebrate Animals

- Not Applicable (No Vertebrate Animals)

Biohazards

- Not Applicable (No Biohazards)

Budget and Period of Support

- Recommend as Requested

PROJECT 3, CRITIQUE 3:

Significance: 6
Investigator(s): 4
Innovation: 4
Approach: 6
Environment: 2

Overall Impact:

The proposed research examines important questions about bidirectional relationships between engagement with self- and other-generated content on social media and adolescent well-being across time, and whether these relations differ for sexual and gender minority youth (SGM) and other youth. Strengths include the direct observation of social media engagement and the focus on SGM youth. This application is limited by serious weaknesses in the conceptualization, few references to previous theory and research that could guide this work, and many questions about how participants will be selected especially the SGM subsample, how social media data will be archived and coded and analyzed, the insufficient measures of well-being, and the analytic plan.

1. Significance:
Strengths

- Examining self- and other-generated social media content in relation to well-being could inform theoretical models and guide interventions.
- Examining bi-directional relationships between social media engagement and well-being across time could inform theoretical models and guide interventions.

Weaknesses

- The conceptual questions posed are general and hypotheses do not focus on specific types of social media content, thus mechanisms explaining relations between social media engagement and well-being cannot really be addressed.
- The application fails to acknowledge earlier research in which social media content has been directly observed.
- The conceptualization of this research is not guided by important theories pertaining to how social media could affect well-being.

2. Investigator(s):
Strengths

- The PI has expertise in the study of TDM of SGM young, and experience in digital ethnography.
- Co-Investigator Toma has expertise in communication science and quantitative methods.

Weaknesses

- The PI is an assistant professor who may not have the necessary experience to lead this project.
- The more senior co-investigators plan to devote little time to this study.

3. Innovation:
Strengths

- The focus on understanding the relations between social media engagement and well-being focus for sexual and gender minority (SGM) youth is novel.
- Although previous investigators have directly observed the content of social media communication by youth, the use of qualitative methods is innovative.

Weaknesses

- For the most innovative part of this study, examining how social media engagement relates to well-being for SGM youth, no specific hypotheses are advanced.
- This application barely acknowledges prior work involving directly observing youth's social media communication, thus does not make clear what is novel about this approach.

4. Approach:

Strengths

- Directly observing the content of young people's self- and other-generated media content is high on ecological validity.
- Investigating bi-directional relationships between self- and other-generated social media content and well-being across time could provide clues as to the direction of the relationships.

Weaknesses

- The application does not describe how exactly SGM youth will be oversampled.
- The application is unclear as to how exactly social media content will be observed: whether individual study staff will follow participants on social media or whether a lab account will follow participants and how exactly social media content will be downloaded and stored for coding.
- Because social media content will be obtained by simply following participants rather than using archiving applications to gain access to social media accounts, it will not be possible to observe or code direct messages on these platforms. This is missing an important opportunity to assess peer connection and social support.
- The data recording and coding procedures are described in very general terms. It is not clear how reliability will be assessed. It is not clear that content to be coded will be related to two important developmental tasks identified in the parent application: identity development and peer connection.
- For gathering data on other-generated social media content, eliminating any peers with private accounts seriously compromises ecological validity.
- The application does not make clear how phenomenological interviews will be conducted, and how responses to be recorded and coded.
- The measures of well-being seem extremely limited.
- The analytic plan is reasonable as far is it goes but is overly general and does not address Specific Aim 3, the most innovative of the goals of the study.

5. Environment:

Strengths

- The institutional environments of the University of Wisconsin, the University of Michigan, and Brown University all seem strong and supportive.

Weaknesses

- The application could be clearer about how this project will benefit from the scientific environments in which this work will be conducted.

Protections for Human Subjects

- Unacceptable Risks and/or Inadequate Protections
- The screenshot included in the application on p. 556 is a compelling example but including it might violate confidentiality.
- The application does not make clear how the social media data will be downloaded and stored, and how confidentiality will be maintained. This is especially important because social media data cannot be de-identified.
- Participants allowing staff or a lab account to follow them on social media and to be listed among their friends could identify them as research participants and thus compromise confidentiality.
- It is not clear how other-generated social media content will be downloaded and coded, and whether it is ethical to analyze others' social media communication without their consent.

Data and Safety Monitoring Plan (Applicable for Clinical Trials Only):

- Not Applicable (No Clinical Trials)

Inclusion of Women, Minorities, and Individuals Across the Lifespan

- Sex/Gender: Distribution justified scientifically
- Race/Ethnicity: Distribution justified scientifically
- For NIH-Defined Phase III trials, Plans for valid design and analysis: Not applicable
- Inclusion/Exclusion of Children under 18: Including ages <18; justified scientifically

Vertebrate Animals

- Not Applicable (No Vertebrate Animals)

Biohazards

- Not Applicable (No Biohazards)

Budget and Period of Support

- Recommend as Requested

THE FOLLOWING SECTIONS WERE PREPARED BY THE SCIENTIFIC REVIEW OFFICER TO SUMMARIZE THE OUTCOME OF DISCUSSIONS OF THE REVIEW COMMITTEE, OR REVIEWERS' WRITTEN CRITIQUES, ON THE FOLLOWING ISSUES:

P**ROTECTION OF HUMAN SUBJECTS: UNACCEPTABLE**

- In Project 2 and 3, Reviewers raised concern about the social media content download and storage and how confidentiality will be maintained**.**

INCLUSION ACROSS THE LIFESPAN PLAN: ACCEPTABLE

SCIENTIFIC REVIEW OFFICER'S NOTES:

INCLUSION OF WOMEN PLAN: ACCEPTABLE

INCLUSION OF MINORITIES PLAN: ACCEPTABLE

INCLUSION ACROSS THE LIFESPAN: ACCEPTABLE

COMMITTEE BUDGET RECOMMENDATIONS: The budget was recommended as requested.

Footnotes for 1 P01 HD109850-01; PI Name: MORENO, MEGAN A.

NIH has modified its policy regarding the receipt of resubmissions (amended applications).See Guide Notice NOT-OD-18-197 at https://grants.nih.gov/grants/guide/notice-files/NOT-OD-18-197.html. The impact/priority score is calculated after discussion of an application by averaging the overall scores (1-9) given by all voting reviewers on the committee and multiplying by 10. The criterion scores are submitted prior to the meeting by the individual reviewers assigned to an application, and are not discussed specifically at the review meeting or calculated into the overall impact score. Some applications also receive a percentile ranking. For details on the review process, see http://grants.nih.gov/grants/peer_review_process.htm#scoring.

MEETING ROSTER

The roster for this review meeting is displayed as an aggregated roster that includes reviewers from multiple HD Special Emphasis Panels Meetings
for the 2022/05 council round.
This roster for HD is available here.
